# Modeling basal body temperature data using horseshoe process regression

**DOI:** 10.1002/sim.9991

**Published:** 2023-12-14

**Authors:** Elizabeth C. Chase, Jeremy M. G. Taylor, Philip S. Boonstra

**Affiliations:** 1 Statistics Group, RAND Corporation, Arlington, VA, USA; 2 Department of Biostatistics, University of Michigan, Ann Arbor, Michigan, USA

**Keywords:** horseshoe prior, local shrinkage, menstrual cycle, nonparametrics, step functions

## Abstract

Biomedical data often exhibit jumps or abrupt changes. For example, women’s basal body temperature may jump at ovulation, menstruation, implantation, and miscarriage. These sudden changes make these data challenging to model: many methods will oversmooth the sharp changes or overfit in response to measurement error. We develop horseshoe process regression (HPR) to address this problem. We define a horseshoe process as a stochastic process in which each increment is horseshoe-distributed. We use the horseshoe process as a nonparametric Bayesian prior for modeling a potentially nonlinear association between an outcome and its continuous predictor, which we implement via Stan and in the R package HPR. We provide guidance and extensions to advance HPR’s use in applied practice: we introduce a Bayesian imputation scheme to allow for interpolation at unobserved values of the predictor within the HPR; include additional covariates via a partial linear model framework; and allow for monotonicity constraints. We find that HPR performs well when fitting functions that have sharp changes. We apply HPR to model women’s basal body temperatures over the course of the menstrual cycle.

## 1 ∣ INTRODUCTION

Consider a longitudinal outcome, such as a man’s prostate specific antigen (PSA) tracked throughout treatment for prostate cancer or a woman’s basal body temperature measured during the menstrual cycle. Common approaches to model these data might include a generalized linear model, splines, or a version of Gaussian process regression, all of which would recover an estimate of the underlying association between time and the outcome, provide a measure of uncertainty about that association, and enable interpolations between observed datapoints, with varying degrees of assumptions, computational speed, smoothness, and interpretability.^1^ However, the examples given above—and many other types of biomedical and scientific data—share a common trait: the possible presence of jumps, kinks, or steps in the association between the outcome and predictor. In the case of PSA and prostate cancer, we might see such a jump after the removal of the prostate, when PSA plummets, and again when radiation therapy is initiated. In between these drops, we would observe more gentle increases in PSA as the prostate cancer progresses.^2^ For women’s basal body temperature, this jump occurs at ovulation, when body temperature increases sharply in response to progesterone release, after which point body temperature will jump or gradually decline back to the pre-ovulation temperature by the end of the menstrual cycle.^3^ The number of jumps and their locations may not be known *a priori*. In the case of PSA monitoring, we would expect to know when the patient received particular treatments, but with women’s basal body temperature, it is less likely that we would know the location of the jump(s) without some examination of the data. An optimal statistical method would recognize the jumps—regardless of whether their locations are known *a priori*—and allow them to be sharp, but without introducing noise in the smooth parts of the association. This is where the methods listed above struggle, failing to accommodate both the sharp jumps and the smooth portions.^4^

This paper studies horseshoe process regression, a method to model data featuring sharp jumps and smooth portions when the locations of the jumps are not known *a priori*. To do so, we adapt Gaussian process regression by using a horseshoe process prior rather than a Gaussian process prior. The horseshoe process prior accommodates large jumps and constant stretches, and uses information from the data to place the jumps. Extensions allow for interpolations and predictions at unobserved datapoints, the inclusion of multiple predictors (both linear and nonlinear), non-Gaussian outcomes, and monotonicity constraints. Because the model is implemented in a fully Bayesian framework, uncertainty estimates are straightforward to calculate. We provide an R package, HPR, which includes all of these features and other functions useful for applied practice.

Horseshoe process regression adds to the extensive literature on change point and step detection modeling, sometimes called mean or trend filtering. Early methods included cumulative sum testing approaches^3^ or running median filters.^4^ Other common approaches include the use of low-degree splines,^4^ often with some kind of penalty,^5^ or LASSO variants.^6^ Gaussian process models, modified to be autoregressive or nonstationary, are another flexible option.^7^ Many sophisticated methods have come from the econometrics literature. These include jump diffusion models, jump processes, and stochastic volatility models.^8,9^ Although these approaches produce very flexible fits, they are often poorly suited to the biomedical setting: performance relies on large numbers of observations, usually equally-spaced, that are rarely available in patient biomarker data, and as a result, overfitting and model nonconvergence can be serious concerns in small or unequally spaced samples. There is little consideration of non-Gaussian outcomes. In addition, because time is often the predictor of interest in these settings, there is no allowance for observations at the same predictor value, as might be seen in the biomedical setting when working with dose-toxicity data, in which the predictor is dose and multiple patients could be assigned to receive the same dose.

Faulkner and Minin^10^ were the first to recognize the potential of horseshoe processes for nonparametric curve fitting in their exploration of Bayesian shrinkage priors for trend filtering on $k^{th}$ order differences, which they call shrinkage prior Markov random fields (SPMRF). They presented evidence that the horseshoe prior was well-suited to piecewise-constant curve estimation within the context of Bayesian trend filtering. Similarly, Kowal et al^11^ developed dynamic shrinkage processes for use in stochastic volatility modeling. Their model formulation resembles ours and SPMRF, but its primary focus is on a dynamically dependent variant of the model. In addition, it is targeted to econometric applications, which limits its use in the biomedical setting, with difficulties with repeated or unequally spaced predictor values. We build upon both Faulkner and Minin^10^ and Kowal et al^11^ by allowing for data interpolation, additional linear predictors, and monotonicity constraints, and the insight we offer into computational performance. Another key building block of our paper is Boonstra et al^12^ work on horseshoe priors for isotonic regression with categorical predictors and binary outcomes. We extend that approach by allowing for continuous predictors through the use of a horseshoe process prior, and we consider continuous and count outcomes in addition to binary outcomes. We also consider data that are not monotonic. Our finished product is a versatile approach for fitting piecewise constant and piecewise linear functions in biomedical settings, with flexible extensions to allow for additional covariates, discrete outcomes, and monotonicity constraints.

The structure of the paper is as follows. First, we present some technical background on the horseshoe prior and the underlying theory of horseshoe processes. Second, we present the model formulation for horseshoe process regression. We review the extensions to allow for data interpolation, additional covariates, and monotonic constraints, and provide computational details. Third, we present a simulation study to characterize horseshoe process regression’s performance. We demonstrate the use of horseshoe process regression in a dataset of women’s basal body temperatures from Weschler.^13^ We conclude with a discussion of limitations and directions for future work.

## 2 ∣ TECHNICAL BACKGROUND

### 2.1 ∣ The horseshoe prior

Consider the classic linear regression problem in which we have observations $y_{i}$ and a length p vector of predictors $x_{i}$ for each individual i=1,…,n. We might wish to fit a multivariable linear regression of the form $y_{i}=\beta_{0}+\beta_{1}x_{1i}+\beta_{2}x_{2i}+\cdots +\beta_{p}x_{pi}+\epsilon_{i}$, $\epsilon_{i}\overset{iid}{∼}N(0,\sigma^{2})$. If p is large—possibly even larger than n—we may suspect that many of the coefficients $\beta_{j}$, j=1,…,p are close to zero, with little effect on the outcome y. Maximum likelihood estimation of the regression is not possible if p>n; even if p≤n, the estimates of the coefficients may be highly variable. Shrinkage estimation is one approach to address this problem, in which we use a penalty or other constraint in model estimation to *shrink* the coefficients closer to zero. Ideally, this shrinkage constraint will push the coefficient estimates for predictors that have little association close to zero while leaving untouched the coefficient estimates for predictors that have a large association with the outcome.^1^

The horseshoe prior is a popular Bayesian approach for shrinkage estimation.^14^ It takes the form $p(\beta_{j}|\tau^{2},\lambda_{j}^{2})∼N(0,\tau^{2}\lambda_{j}^{2})$, with $\tau ∼C^{+}(0,c)$ and, independently, $\lambda_{j}\overset{iid}{∼}C^{+}(0,1)$, where $C^{+}(a,b)$ denotes a half-Cauchy distribution with location parameter a and scale parameter b. We call τ the *global shrinkage parameter*, as it provides an overall measure of shrinkage on the $\beta_{j}’s$. If τ is large, this prior admits many large $\beta_{j}’s$; if τ is small, the $\beta_{j}’s$ are pushed towards zero. However, the horseshoe prior also contains a set of *local shrinkage parameters*, $\lambda_{j}$, j=1,…,p, one for each $\beta_{j}$. The local shrinkage parameters allow individual $\beta_{j}’s$ to attain high values, even if τ is small. The marginal density function of the horseshoe prior takes on a distinctive shape, featuring an infinite spike at zero, along with moderately heavy tails. As a result, the horseshoe distribution approaches zero faster than a normal distribution, but while admitting large values with high probability. It has been shown that separately for each coefficient, the horseshoe favors either total shrinkage (estimate of $\beta_{j}$ close to zero) or minimal shrinkage (leaving the estimate of $\beta_{j}$ close to its estimate under maximum likelihood estimation).^14^

The horseshoe prior provides excellent shrinkage performance and resolves many of the computational difficulties of earlier Bayesian approaches to shrinkage estimation.^14,15^ However, it has drawbacks: the heavy tails of the half-Cauchy priors on τ, $\lambda_{j}$ can cause problems with Bayesian model convergence. Proposed solutions to these problems include the use of decentered parameterizations to reduce *a priori* parameter correlation;^16^ imposing additional regularization on the tails of the half-Cauchy priors;^17^ and placing an additional hyperprior on the scale parameter c of the prior on τ.^18^

### 2.2 ∣ Horseshoe processes

We will now switch focus to stochastic processes. Suppose y(t) is some outcome recorded at time t. We use time for simplicity, but note that t could be any continuous predictor. In its simplest form, a Gaussian process is defined as $y(t)-y(t-s)∼N(0,s\tau^{2})$. A Gaussian process assumes that incremental change over time is normally distributed. It relies on a single parameter, $\tau^{2}$, which is the variance of the process. If $\tau^{2}$ is large, the process will vary substantially over time; if $\tau^{2}$ is small, the process will remain fairly constant over time. The variance between measurements increases as the elapsed time between them, s, increases.

We seek to define a horseshoe process, which takes a similar structure to a Gaussian process, except incremental change over time will be horseshoe distributed rather than normally distributed. Polson and Scott^19^ demonstrated that all local-global shrinkage distributions based on scale mixtures of normals (like the horseshoe) can be extended to a stochastic process within the framework of subordinated Brownian motion. They found that a horseshoe process H(t) can be represented as H(t)∼N(0,exp(M(t))) where M(t) is a Meixner process. Although this definition of a horseshoe process is the most mathematically complete, obstacles lie in the way of implementing it. The Meixner process has few workable computational implementations.^20^ In addition, because the Meixner-subordination occurs on the log-variance scale for the horseshoe process (rather than the variance scale), it is challenging to understand the covariance structure and other properties of the horseshoe process.^19^ In light of these challenges, we use a discrete formulation of the horseshoe process. For a set of timepoints $t_{k}$, k=1,…,m, define a horseshoe process $H_{t_{k}}$ as:

(1) $$H_{t_{k}}-H_{t_{k-1}}|\tau ,\lambda_{k}∼N(0,\tau^{2}\lambda_{k}^{2}(t_{k}-t_{k-1})),\;k=2,\ldots ,m\;\;H_{t_{1}}=0\;\;\tau ∼C^{+}(0,c)\;\;\lambda_{k}\overset{iid}{∼}C^{+}(0,1),k=2,\;\ldots \;,m$$

Under this formulation, incremental motion is horseshoe distributed, defined only at discrete observations of continuous time. Each discrete time increment has its own local shrinkage parameter, $\lambda_{k}$, while the overall variance is controlled by the global shrinkage parameter, τ. Variance between observations continues to scale with elapsed time.

This discrete definition of a stochastic process may pose theoretical challenges, for example, what is the value of $H_{t^{*}}$ for $t_{k-1}<t^{*}<t_{k}?$ For a Gaussian process this is readily obtained, but because of the local shrinkage parameters of the horseshoe process, it is more challenging. $H_{t^{*}}$ must have its own local shrinkage parameter. The only way to truly resolve this difficulty is by pursuing the Meixner-subordinated process described above, in which we have a continuously generated stochastic process of local shrinkage parameters. However, theoretical and computational development of that model remains intractable.

In the interim, one solution might be to define the value of the local shrinkage parameter to be some fixed value within each increment. This approach is somewhat unsatisfactory, though, because the carried-forward local shrinkage parameter approach blunts the horseshoe process’ most unique feature: its abrupt, dynamically changing behavior with the rapidly changing local shrinkage parameters. Rather than define the local shrinkage parameter of $H_{t^{*}}$ to be some function of the other local shrinkage parameters, we instead use the Bayesian imputation scheme described below, and impute a value for the missing local shrinkage parameter for any unobserved values of the horseshoe process at which predictions are desired. Therefore, the “horseshoe process” is only defined for discrete observations—but a horseshoe process can be generated for *any* set of predefined times.

## 3 ∣ METHODS

### 3.1 ∣ Model formulation

Let $y_{i}$ be some outcome observed for patients i=1,…,n at continuous predictor value $x_{i}$. Define x, y as the corresponding length n vectors of these observations. Define t as a length m vector containing the unique, ordered values of x. Suppose that $x_{i}=t_{j}$. Then we define a horseshoe process regression (HPR) model as:

(2) $$g(E(y_{i}))=f_{j}=\alpha +\sum_{k=1}^{j}h_{k}\;h_{k}|\tau ,\lambda_{k}∼N(0,\tau^{2}\lambda_{k}^{2}(t_{k}-t_{k-1})),\;k=2,\;\ldots \;,m,\;h_{1}=0\;\;\tau ∼C^{+}(0,c),\;\lambda_{k}\overset{iid}{∼}C^{+}(0,1),\;k=2,\;\ldots \;,m\;\;\alpha ∼N(a,b^{2})$$

In (2), we assume that f, the appropriately-transformed mean trajectory of y with respect to time, follows a horseshoe process, formulated as the sum of the discretely observed horseshoe increments. This corresponds to placing a horseshoe prior on the first order differences of f and approximates placing a horseshoe prior on the first derivative of the association. The HPR model assumes that the functional form is like a step function. In general, the $\lambda_{k}$ values will be small, resulting in long stretches of near-constant values of f, punctuated by abrupt steps which may be quite large when large values of $\lambda_{k}$ are supported by the data. The process starts at a y-intercept α, which has a normal prior placed on it. The $g(E(y_{i}))$ formulation allows for non-Gaussian data through the use of an appropriate transformation g. We use a logit transformation and Bernoulli likelihood for binary outcomes and a log transformation and Poisson likelihood for count data. The model also allows for multiple observations at the same $t_{j}$ value and does not require the t to be evenly spaced.

### 3.2 ∣ Extension 1: Interpolation and prediction

As is often the case, there may be values of x at which we wish to obtain predictions or extrapolations, or to obtain a more finely-spaced grid of increments $h_{k}$ with which to approximate the horseshoe process.^21^ In this case, we can augment the grid t with additional gridpoints that are not included in x so that t is the unique, ordered set of observed x values and augmentation points $x_{aug}$. Let $y_{obs}$ denote the observed values of y, and let $f_{obs}$ denote the estimates of the underlying transformed mean of y at the observed locations. Define $y_{aug}$, $f_{aug}$ as the unobserved outcome and transformed mean at the requested augmentation points. Let θ represent the hyperparameters of the model, for example, θ={α,h,λ,τ}. Then we wish to obtain samples from the posterior distribution of $f_{obs}$, $f_{aug}$, θ.^22^ A common approach to do this would be to place a prior on $y_{aug}$ to reflect the additional uncertainty for the imputed outcomes, for example, $y_{aug}∼N(f_{aug},\sigma^{2})$ in the case of Gaussian outcomes. However, in the case where we only care about the underlying mean trajectory $f_{aug}$, we see that placing a prior on $y_{aug}$ is unnecessary, because it does not contribute to the posterior of the other parameters:

(3) $$P(f_{obs},f_{aug},\theta |y_{obs})\propto \int P(f_{obs},f_{aug},y_{obs},y_{aug},\theta )dy_{aug}\;\;=\int P(y_{obs}|f_{obs},\theta )P(y_{aug}|f_{aug},\theta )P(f_{obs},f_{aug},\theta )dy_{aug}\;\;=P(y_{obs}|f_{obs},\theta )P(f_{obs},f_{aug},\theta )$$

Then for an unobserved element of t, we sample a corresponding value of f according to some prior distribution for $f_{aug}$, without needing to specify a prior distribution for $y_{aug}$. We choose to impose the same form on $f_{aug}$ as on $f_{obs}$, for example, an augmentation point $x^{*}=t_{j}$ will have $f_{j}=\alpha +\sum_{k=1}^{j}h_{k}$ with its own local shrinkage parameter $\lambda_{j}$ sampled, where $\lambda_{j}∼C^{+}(0,1)$. Note that this approach assumes that $f_{\mathrm{aug}}$ and $f_{\mathrm{obs}}$ have a similar level of abrupt change. For example, if at the observed datapoints $f_{\mathrm{obs}}$ appears relatively flat, but at the augmentation points $f_{\mathrm{aug}}$ is abruptly changing, then the above augmentation approach will not be able to interpolate those jumps in the association because there is no evidence of them in the observed data. If data are observed at particular times/locations because they are more or less variable than at unobserved times/locations–for example, some biomarker that is deliberately measured in response to low or high levels of variability–then our approach will produce biased estimates of $f_{\mathrm{aug}}$. However, the estimates of $f_{\mathrm{obs}}$ should be unaffected.

This approach highlights some of the limitations of working with a discrete formulation of a horseshoe process. Every unique value of t must have its own local shrinkage parameter, whether there is an observed outcome at that location to support its estimation or not. In a truly continuous formulation of a horseshoe process, we would have posterior estimates of the parameters of the underlying Meixner process that generated the local shrinkage parameters. Thus–in an ideal world–we might be able to obtain the posterior distribution of $\lambda_{\mathrm{aug}}|\lambda_{\mathrm{obs}},y_{\mathrm{obs}}$ from the properties of the multivariate Meixner distribution, and then use these values in combination with properties of the multivariate normal distribution to obtain the distribution of $f_{\mathrm{aug}}|y_{\mathrm{obs}}$, possibly without needing to rerun Markov Chain Monte Carlo (MCMC) sampling.^23^ The proposed Bayesian imputation approach, which samples values of λ at the augmentation points, may be more computationally burdensome, and we might worry about instability in the presence of large numbers of augmented datapoints or large gaps between them. The simulation study conducted below is meant to address some of these concerns.

### 3.3 ∣ Extension 2: Partial linear models

We can extend (2) to include multiple predictors using a partial linear model framework. Suppose we have a length p vector of covariates $z_{i}$ for each subject, yielding an n×p matrix Z of covariates. If we assume that these predictors are linearly related to the outcome y, then we can extend the model to be:

(4) $$g(E(y_{i}))=f_{i}=\alpha +\sum_{k=1}^{j}h_{k}+\beta z_{i}\;\;\beta ∼N(0,d^{2})$$

with all other priors unchanged and again assuming that $x_{i}=t_{j}$. We now include the parameters β, a length p vector of coefficients, each of which have a normal prior with mean 0 and variance $d_{l}^{2}$, l=1,…,p. Z can contain either continuous or categorical predictors; categorical predictors would need to be converted to a dummy parameterization. We could also consider different priors on β such as a Cauchy prior rather than a normal prior if more regularization is desired.^24^ It may also be possible to include multiple horseshoe smoother terms in an additive formulation, although this is not something we explore here.^11^

### 3.4 ∣ Extension 3: Monotonicity constraints

In some settings, we may wish to constrain the HPR to be monotonic, that is, for $t_{j}<t_{k}$, $f_{j}\leq f_{k}$ for monotonic increasing. Monotonicity constraints are easily introduced via a transformation of the first derivative to constrain it to be nonnegative (or nonpositive). We propose using the absolute value function as our transformation.^12^ Then we modify the HPR to be:

(5) $$g(E(y_{i}))=f_{j}=\alpha +\sum_{k=1}^{j}|h_{k}|$$

All priors are unchanged from before; the only difference is that the horseshoe process sums the transformed increments. As a result, the first derivative is constrained to be nonnegative, and thus the function must be monotonic increasing. (We could similarly take $-|h_{k}|$ to obtain a monotonic decreasing function.)

### 3.5 ∣ Computation

There are several parameters and hyperparameters whose priors merit further discussion. We recommended placing a normal prior with mean a and variance $b^{2}$ on α, the y-intercept of the HPR. We recommend setting a to be $\bar{y}$, the sample mean of y and b to be five times $sd_{y}$ the sample standard deviation of y. When linear predictors are present, unless there is subject knowledge to further refine prior specifications, we recommend centering and scaling all continuous predictors to have a scale of 1 for continuous outcomes and 0.5 for discrete outcomes, and then setting the prior scale on the linear coefficients to be 5 for continuous outcomes and 2.5 for discrete outcomes.^25^

The scale parameter of the global variance, c, is also important. Some may choose to place a hyperprior on c that favors small values, such as an inverse-Gamma or a tightly-constrained half-normal distribution. In our experience, the value of c has little effect on the resulting HPR fit unless data are very sparse. For all models, we set it to be equal to 0.01, which we find yields good performance. However, future work may wish to investigate this further in the sparse data setting.

We implement all models using Hamiltonian Monte Carlo (HMC) via Stan and the cmdstanr package in R. Rather than explore the parameter space through random steps, HMC uses Hamiltonian dynamics to guide movement through the parameter space.^26^ As implemented in Stan, HMC is combined with the No-U-Turn-Sampler (NUTS), which assists with HMC’s tuning and provides additional diagnostics.^27^ To interface Stan with R, we use the cmdstanr package.^28^

In complex models like ours, the performance of HMC is affected by the choice of model parameterization. Decentered parameterizations are favored, in which no priors feature other parameters, to reduce *a priori* dependence between parameters. Relationships between parameters are instead obtained through transformations that are performed after parameter sampling steps.^16^ We use decentered parameterizations for all parameters in the model. Thus, the full list of parameters and priors which we sample is:

-
$\alpha ∼N(\bar{y},{\left(5sd_{y}\right)}^{2})$

- $\tau =c\tau_{1}\sqrt{\tau_{2}}$, where $\tau_{1}∼N(0,1)$ and $\tau_{2}∼\mathrm{InvGamma}\left(\frac{1}{2},\frac{1}{2}\right)$. Note that this corresponds to $\tau ∼C^{+}(0,c)$.
- Independently for k=2,…,m, $\lambda_{k}=\lambda_{1k}\sqrt{\lambda_{2k}}$, where $\lambda_{1k}∼N(0,1)$ and $\lambda_{2k}∼\mathrm{InvGamma}\left(\frac{1}{2},\frac{1}{2}\right)$. Note that this corresponds to $\lambda_{k}∼C^{+}(0,1)$.
- For k=2,…,m, $h_{k}=\gamma_{k}\tau \lambda_{k}\sqrt{\left(t_{k}-t_{k-1}\right)}$, where $\gamma_{k}∼N(0,1)$. Note that this corresponds to $h_{k}|\tau$, $\lambda_{k}∼N(0,\tau^{2}\lambda_{k}^{2}(t_{k}-t_{k-1}))$.
- In the case of Gaussian outcome data: $\sigma =\sigma_{1}\sqrt{\sigma_{2}}$, where $\sigma_{1}∼N(0,s^{2})$ and $\sigma_{2}∼\mathrm{InvGamma}\left(\frac{1}{2},\frac{1}{2}\right)$. Note that this corresponds to $\sigma ∼C^{+}(0,s)$. In the simulations below, we use s=5.
- In the case of linear covariates: a length p vector $\beta ∼N(0,d^{2})$.

After making necessary parameter transformations, we connect these parameters to the data using an appropriate likelihood (Gaussian, Bernoulli, or Poisson).

For HMC sampling, we use four chains, each with a warm-up phase of 1000 samples and a sampling phase of 2000 samples, without thinning. This yields a total of 8000 samples from the posterior distribution. Despite the efforts described above to improve computational tractability, it is still common to observe occasional HMC divergences, which usually indicate poor posterior exploration. We present evidence that these sporadic divergences do not seem to harm model performance, although high numbers of divergences should still be cause for concern.^26^

All of the methods described above are implemented in the R package HPR, hosted on GitHub.^29^ The package is used to conduct the simulation studies described below and the data application.

## 4 ∣ SIMULATION STUDY

### 4.1 ∣ Horseshoe process regression

We considered four true underlying associations, each of which were observed at an equally spaced grid of n=100 observations:

1. bigstep: f(x)=0∗I(x≤2)+6∗I(2<x≤5)+1∗I(5<x≤6)+3∗I(6<x≤8)+10∗I(x>8)
2. joinpoint: f(x)=(1.5x)∗I(x<2)+(16−5x)∗I(2≤x<3)+1∗I(3≤x<6)+(10−x)∗I(6≤x<9)+(5x−44)∗(x≥9)
3. impulse: f(x)=0∗I(x=0)+exp(−x)∗I(0<x<3)+1∗I(x=3)+exp(−(x−3))∗I(3<x<7)+exp(−(x−7))∗I(x=7)
4. bounce: f(x)=∣sin(x)∣

**FIGURE 1:**
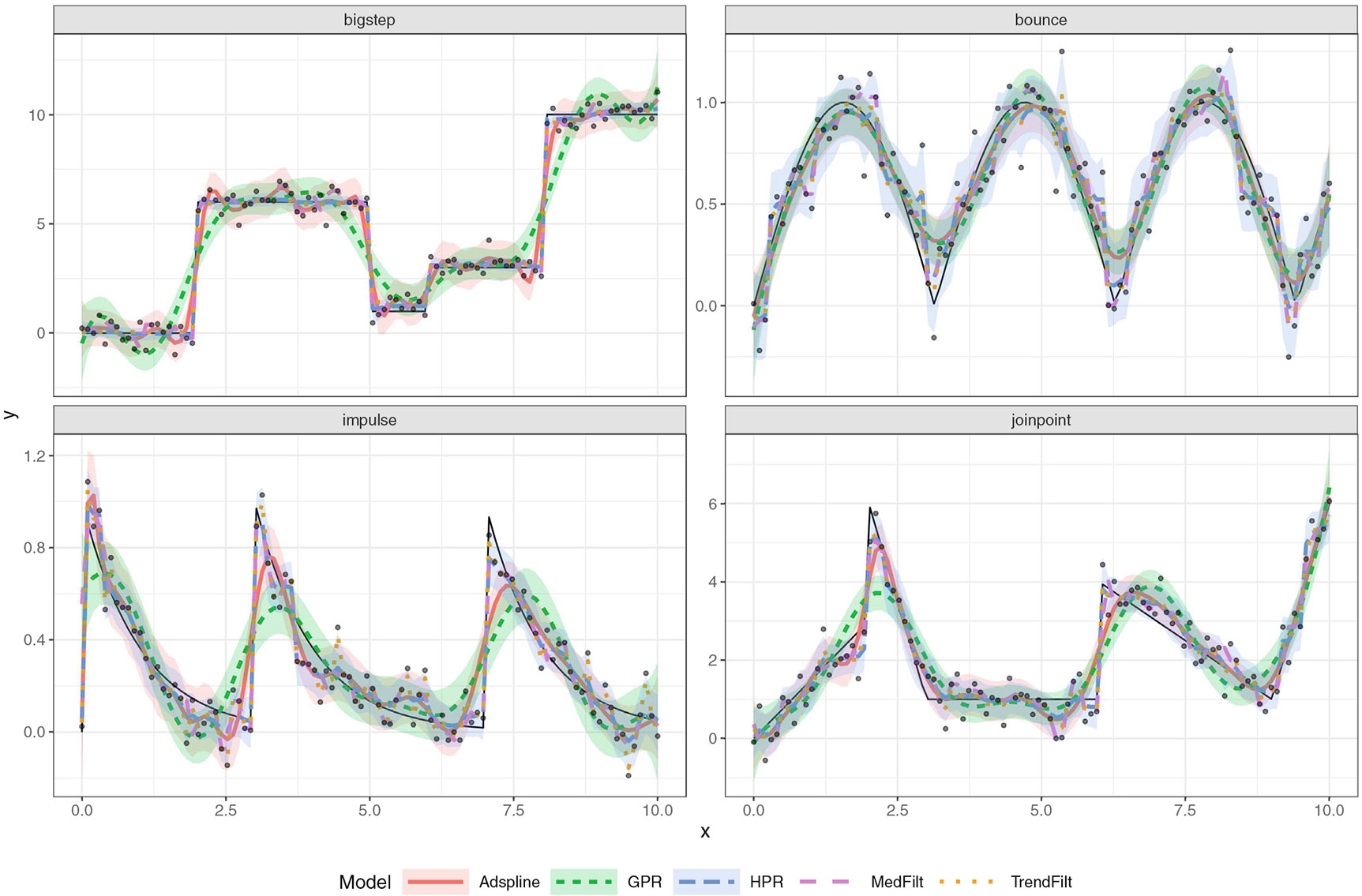
Point estimates and 95% credible/confidence intervals of horseshoe process regression (HPR) and comparison methods on sample datasets from four data-generating scenarios for continuous outcomes, each with *n* = 100. Comparison methods are an adaptive spline model (Adspline), a Gaussian process regression (GPR), a median filter (MedFilt), and a penalized trend filter (TrendFilt). A color version of this figure is available online.

I() denotes the indicator function; that is, I(x)=1 if condition x is true, and I(x)=0 otherwise. For the Gaussian outcomes, we simulated measurement error with σ=0.5 for the bigstep and joinpoint scenarios, σ=0.2 for the bounce scenario, and σ=0.1 for the impulse scenario. Plots of the true underlying curves along with a sample dataset are given in Figure 1 for continuous outcomes. Based on the underlying model, we expected HPR to excel at the bigstep scenario. Joinpoint and impulse both featured abrupt changes, but not of HPR’s assumed form, so it was less clear how HPR would perform in these settings. We anticipated weak performance in the bounce scenario, which was primarily smooth and without abrupt changes, but we included it to provide insight into HPR’s limitations. We considered a number of comparison methods. First, we compared to Gaussian process regression (GPR), a smooth approach which places a Gaussian process prior on the function fit, using the R package mgcv.^30^ Second, we compared to an adaptive smoother (Adspline), which uses the set of unique x values as knot locations for a set of P-splines, which are adaptively penalized.^30^ Third, we compared to the running median filter (MedFilt) as implemented in the package FBN, which estimates the function at x as the median of some number of observations neighboring x. We set a window-size of 3, meaning that the function at x is estimated as the median of y(x) and its immediate neighbors on either side.^4^ Fourth, we compared to the first-order trend filter of Tibshirani et al (TrendFilt) from the package genlasso,^6^ which penalizes the first order differences of the function, and sets the penalty parameter using generalized cross validation. Note that MedFilt and TrendFilt do not provide uncertainty quantification and thus their performance on metrics like credible interval coverage and width is not presented.

We assessed performance with three metrics:

1. Mean absolute difference (MAD): $\frac{1}{n}\sum_{i=1}^{n}|f(x_{i})-\hat{f}(x_{i})|$, where $\hat{f}(x_{i})$ is the predicted function’s value at $x_{i}$ and $f(x_{i})$ is the true function’s value at $x_{i}$.
2. Credible/confidence interval width (Width): $\frac{1}{n}\sum_{i=1}^{n}\hat{f}{\left(x_{i}\right)}^{0.975}-\hat{f}{\left(x_{i}\right)}^{0.025}|$, where $\hat{f}{\left(x_{i}\right)}^{0.975}$ denotes the upper bound of a 95% credible/confidence interval for $\hat{f}(x_{i})$ and $\hat{f}{\left(x_{i}\right)}^{0.025}$ is the lower bound.
3. Credible/confidence interval coverage (Coverage): $\frac{1}{n}\sum_{i=1}^{n}I\left(\hat{f}{\left(x_{i}\right)}^{0.025}\leq f(x_{i})\leq \hat{f}{\left(x_{i}\right)}^{0.975}\right)$.

Note that these three metrics were summed across all of the observed datapoints. We present additional results on pointwise performance in the Supporting Information, Section 2. We assessed performance on each metric across the 100 replicates of each of our four data-generating scenarios for each method. All simulations were implemented in R, using tools from the dynamic statistical comparisons (DSC) framework.^29,31^ All code used to completely reproduce the simulations can be found on GitHub.

**FIGURE 2:**
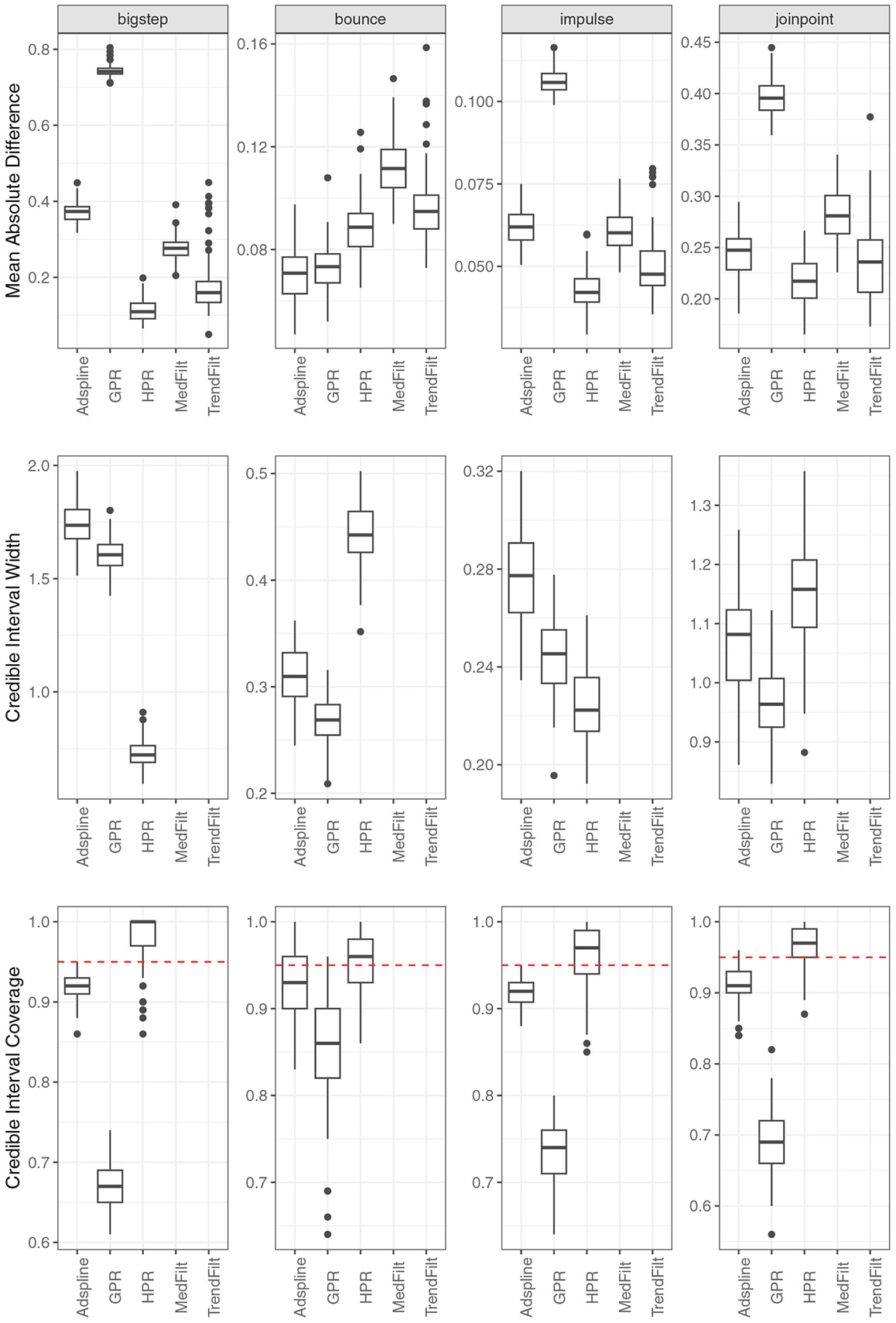
Horseshoe process regression (HPR) simulation results for continuous outcomes, based on 100 replicates on four data-generating scenarios, each with *n* = 100. Comparison methods are an adaptive spline model (Adspline), a Gaussian process regression (GPR), a median filter (MedFilt), and the penalized trend filter (TrendFilt). The top row gives performance for mean absolute difference (smaller is better); the second row gives performance for credible/confidence interval width; the third row gives performance for credible/confidence interval coverage (0.95 is nominal and given as a horizontal red dashed line). Each column is for one data-generating scenario; sample datasets for each scenario are shown in Figure 1. Note that interval coverage/width is not given for MedFilt and TrendFilt because these methods do not provide uncertainty estimation. A color version of this figure is available online.

Results for continuous outcomes are given in Figure 2. As we can see, HPR performed quite well, with the smallest MAD of any of the comparison methods for the bigstep, impulse, and joinpoint scenarios. As expected, for the bounce scenario, it was surpassed by the methods that are better-suited to smooth associations, although it provided better results than the other non-smooth methods (TrendFilt, MedFilt). Its credible interval width was slightly wider than the width of the intervals returned by the other methods that give uncertainty estimates, with the exception of the bigstep and impulse scenarios, for which HPR returned a substantially narrower credible interval across the domain than the other comparison methods. Coverage was generally good, with rates above 95% for all of the scenarios. When we examine the pointwise plots in the Supplement (Figure S5), we see that the pointwise performance resembled that averaged over the curve. In particular, the comparison methods particularly struggled at the jumps in bigstep, joinpoint, and impulse, as we expected, with bias spiking at the jump points while credible interval coverage dropped.

Additional results are given in Supplemental Sections 1–3 for count outcomes (Figures S1, S2, and S6), binary outcomes (Figures S3, S4, and S7), and monotonicity constraints (Figures S8–S10). Performance was generally similar to that of continuous data. HPR continued to offer the best performance of all comparison methods for fitting step functions. For binary outcomes, though, GPR offered better performance than HPR for the joinpoint scenario in terms of MAD (Figure S4). Coverage was nominal except for the bigstep scenario, for which it was less than nominal for binary and count outcomes–although still closer to nominal than the other comparators.

### 4.2 ∣ Data interpolation and prediction

Here, we focus on our data interpolation scheme, to assess whether it performs sensibly at varying grid densities. We only considered scenarios bigstep and bounce, and we restricted our focus to HPR, as the data interpolation performance of the comparison methods from section 4.1 have been assessed elsewhere.^6,30^ We randomly sampled 100 unevenly spaced datapoints between 0 and 10 to be our observed x locations. Then, we fit the HPR either (1) only using the observed datapoints, (2) augmented by a grid of datapoints at every 0.5 (21 augmented datapoints), and (3) augmented by a grid of datapoints at every 0.1 (101 augmented datapoints). Note that for some replicates the augmented datapoints will be extrapolations, because we did not require the inclusion of 0 and 10 in our randomly sampled observed data locations. We calculated the performance metrics above separately for the observed datapoints and the augmented datapoints, to see if predictions at the observed datapoints changed depending on the number of gridpoints, and if predictions at the augmented datapoints were fairly accurate.

**FIGURE 3:**
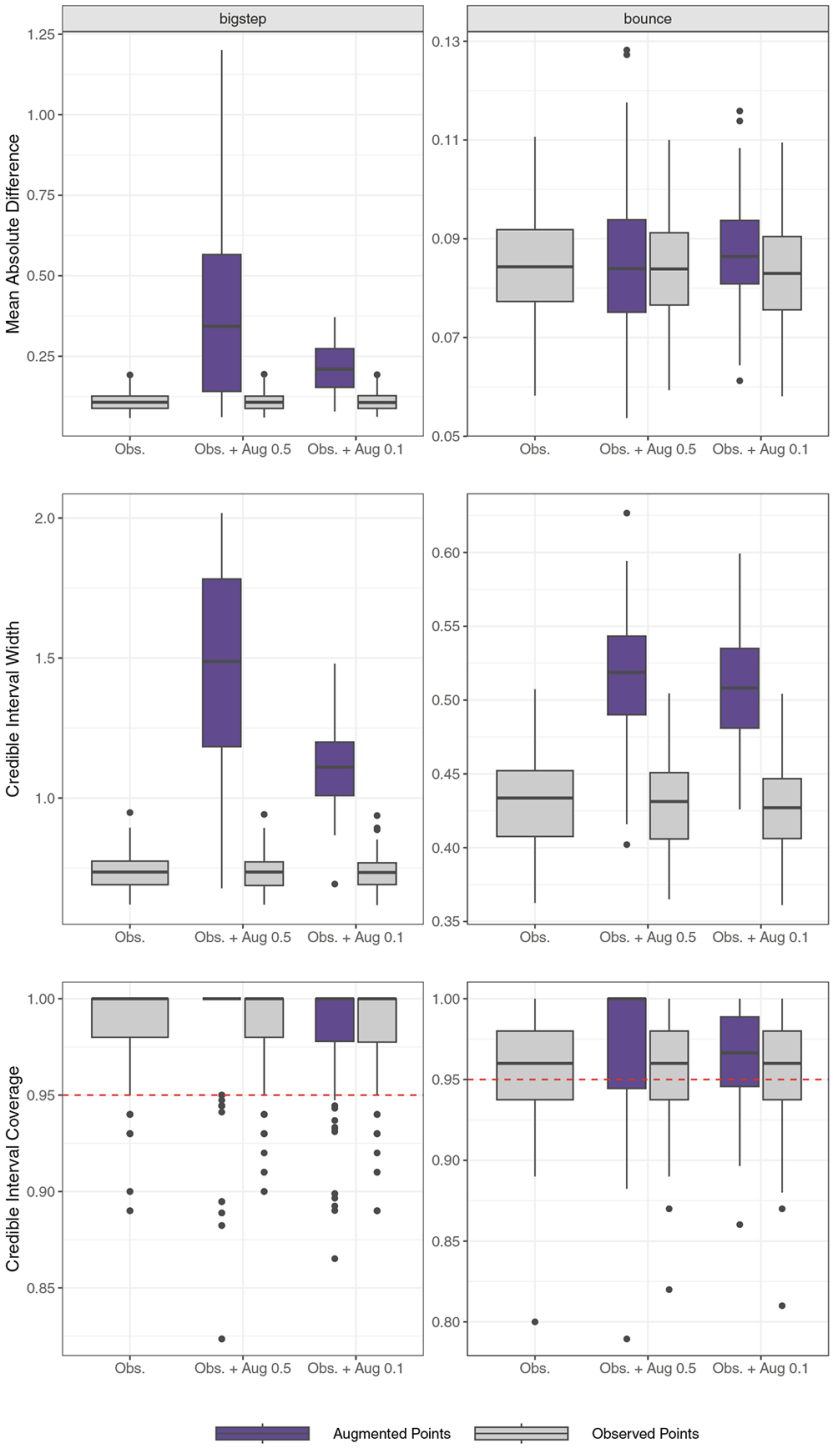
Horseshoe process regression (HPR) data augmentation simulation results for continuous outcomes, based on 100 replicates on two data-generating scenarios. We compared an HPR calculated only at *n* = 100 observed points (Obs) to an HPR with augmentation points at a grid of every 0.5 (Obs + Aug 0.5) and an HPR with augmentation points at a grid of every 0.1 (Obs + Aug 0.1) from 0 to 10. The top row gives performance for mean absolute difference calculated at both the observed and augmented points (smaller is better); the second row gives performance for credible interval width calculated at both the observed and augmented points; the third row gives credible interval coverage calculated at both the observed and augmented points (0.95 is nominal and marked as a horizontal red dashed line). Performance at observed points and augmented points are displayed separately. Each column is for one data-generating scenario. A color version of this figure is available online.

Results for continuous outcomes are given in Figure 3; results for count and binary outcomes are given in Section 4 of the Supplement (Figures S11 and S12). Overall, we see that performance of the augmentation scheme was good, with MAD holding fairly constant, as expected, across observed and augmented datapoints regardless of grid density. MAD was slightly worse at augmented points relative to observed datapoints, and credible interval widths were wider at augmented datapoints than at observed datapoints, as we expected. Both MAD and credible interval width appeared somewhat improved with a larger number of augmentation points. This “improved performance” is misleading, because in the data generating schemes considered here–which do not feature an extremely large number of abrupt changes–the probability that an augmentation point is placed at the location of an abrupt jump is reduced in the presence of more augmentation points, artificially boosting aggregate performance. Credible interval coverage held fairly constant across grid density, with rates at or above 95% for all scenarios. Coverage rate was similar across observed and augmented datapoints. Performance was generally similar for binomial and Poisson outcomes.

### 4.3 ∣ Partial linear models

We conducted simulations to assess the performance of the HPR partial linear model. Full simulation set-up details and results are given in Section 5 of the Supporting Information. Performance of the partial linear model was generally good. HPR offered substantially reduced mean absolute difference and credible interval width for the latent mean $\hat{E}(y_{i})$ when the nonlinear component of the partial linear model was a step function. When the nonlinear component was a smooth function, HPR’s performance was worse than the comparison methods for continuous outcomes, although credible interval coverage was still very good (Figure S13). For binary and count outcomes, HPR consistently surpassed the comparison methods, even when the nonlinear component of the partial linear model was a smooth function (Figures S15 and S17). The Gaussian process regression (GPR) particularly struggled for count outcomes (Figure S15). Regardless of the form of the nonlinear component, performance for estimating the linear effects of the partial linear model was good (Figures S14, S16, and S18).

### 4.4 ∣ Computational assessment and sensitivity analyses

We provide some insight into HPR’s computational performance, with results given in Section 6 of the Supporting Information (Figures S19–S21). Almost all of the models fitted in the simulation studies featured at least some HMC divergences. In most cases less than 5% of samples ended in a divergence. Max treedepth warnings occurred rarely. $\hat{R}$ diagnostics and effective sample sizes generally seemed adequate.^32^ Although slow compared to non-Bayesian methods, computation time was generally quite reasonable, with most models finishing in less than 5 min. This can be made faster with parallelization, which is available in our R package. Models took longer to run as the sample size and the amount of augmentation data increased, as we would expect. For more information on these diagnostics, please see the Stan reference manual.^33^

We also explored the role of sample size and prior specification in model estimation, with full results given in Section 7 of the Supporting Information (Figures S22 and S23). We focused these sensitivity analyses on the bigstep scenario described above, because it is HPR’s recommended setting. In addition to the sample size of n=100 that we used above, we also considered n=30 and n=500. We considered several different settings for the hyperparameters of the model (the prior mean and variance for the y-intercept α, the prior scale c on the global shrinkage parameter τ, and the prior scale s on the measurement error σ). Findings were generally stable across hyperparameter values, although at smaller sample sizes (n=30), findings were more affected by hyperparameter choices. Poor choices for the prior variance on α–particularly setting it too small–negatively affected model fit. The choice of c also affected findings at small sample sizes, especially for binary outcomes. Model estimation improved with larger sample sizes, although estimation was still adequate at the n=30 sample size.

## 5 ∣ APPLICATION

We use HPR to fit the trajectories of women’s basal body temperature (BBT) over the menstrual cycle. BBT follows a reliable pattern in healthy, premenopausal women who are not taking hormonal birth control. Each menstrual cycle starts with the onset of the period, at which time BBT is low. With ovulation (usually around day 14 of the menstrual cycle), a woman’s BBT spikes, sometimes by a full degree Fahrenheit, and will remain high until the onset of the next period, when it will drop suddenly to the pre-ovulation temperature and the cycle will repeat. If the woman conceives a pregnancy that cycle, her BBT will instead stay at the post-ovulation high temperature for the duration of her pregnancy, sometimes even increasing in a second spike around a week after conception when the embryo implants in the uterine lining.

Although this general pattern is consistent across healthy women (sustained low temperature, spike at ovulation, sustained high temperature, drop with onset of period, repeat unless pregnant), the details vary considerably between women and even within a single woman. The date of ovulation may be as early as day 9 or as late as day 30, depending on factors like stress and other medical conditions, with the full menstrual cycle length varying from 20 to 40+ days. Some women exhibit a more gradual increase/decrease in BBT over the menstrual cycle, rather than sharp jumps and drops. However, by tracking BBT over several menstrual cycles, patterns may emerge that suggest underlying health conditions or provide guidance on how best to time sexual intercourse to improve or reduce chances of pregnancy. When used in combination with other health and fertility indicators, BBT plots can provide insight into women’s health.

Here, we model BBT trajectories for several women, using data abstracted from example charts given in Toni Weschler’s *Taking Charge of Your Fertility*.^13^ Weschler presents example plots and gives her hypothesis for the day of ovulation for each plot, based on BBT and other predictors (cervical mucus and position, pregnancy testing information, etc.). We use Weschler’s hypothesis as a best guess for the correct answer, and thus explore HPR’s ability to match the results obtained from careful examination by a trained expert. In all cases, day 1 of the menstrual cycle is the day the period started, and the last observation corresponds to the day of the start of the next period (except in the plots of pregnant women, which we note). We indicate Weschler’s hypothesis on each plot. Our outcome is BBT and our predictor is day of the menstrual cycle. We fit a horseshoe process regression (HPR), and for comparison, a Gaussian process regression (GPR), an adaptive spline model (Adspline), a penalized first-order trend filter (TrendFilt), and a median filter with a window size of 3 (MedFilt). Note that there is no information sharing across women; models are refit separately for each woman’s BBT trajectory.

**FIGURE 4:**
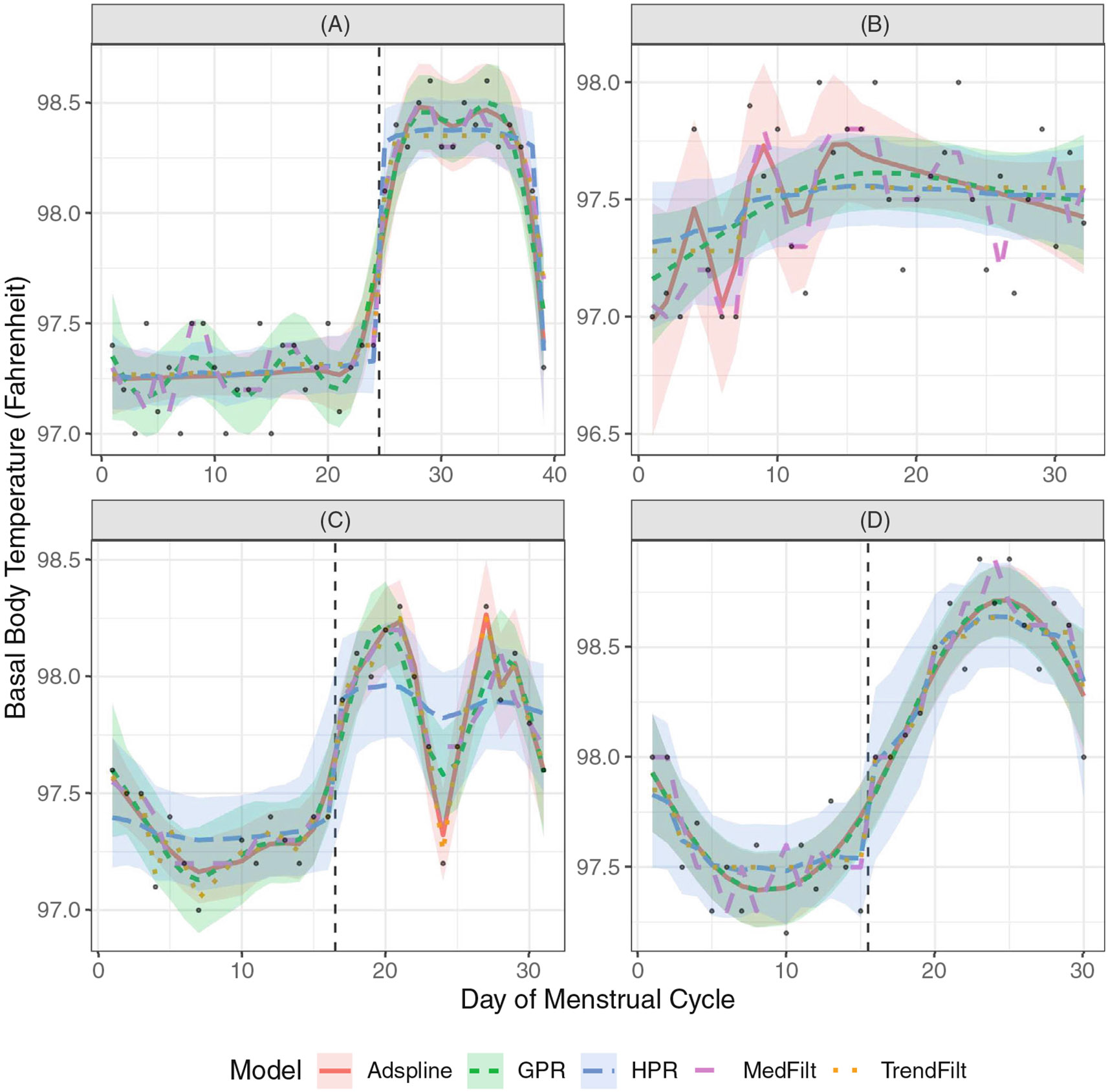
Fitted basal body temperature trajectory and 95% credible/confidence intervals from a horseshoe process regression (HPR), adaptive spline model (Adspline), Gaussian process regression (GPR), median filter (MedFilt), and penalized trend filter (TrendFilt) for four women who did not conceive pregnancy. Observed datapoints are given as black dots, with an expert’s guess of the ovulation time given by the vertical black dashed line. Note that there is no information sharing across women; models are refit separately for each woman’s BBT trajectory. A color version of this figure is available online.

In Figure 4, we present the plots for four women who did not conceive a pregnancy that cycle. In panel A, we see a fairly normal pattern and observe that HPR and TrendFilt both capture the ovulation jump cleanly (although only HPR is able to provide uncertainty estimates). Note that ovulation occurred later than we would expect, at day 24 (with increased temperatures starting on day 25). GPR, by comparison, oscillates substantially in the pre-ovulation phase and over-smoothes the ovulation jump, starting it almost 3 days early. Adspline manages to smooth the pre-ovulation phase, but not the post-ovulation phase, and it also starts the ovulation jump 2 days early. In panel B, we observe no clear pattern—this subject actually did not ovulate that cycle, which is why there is no BBT shift. HPR correctly does not detect one. In panel C, we see another fairly typical example, this one with a slight temperature drop post-ovulation around day 24, which occurs in some women and is normal. Of the three methods, HPR does the best job of capturing the ovulation jump at day 16 without being overly swayed by the minor temperature dip at day 24, while the other methods oscillate heavily in response. Finally, in panel D we see a plot from a woman with weak thermal shift, in that her BBT increases less abruptly with ovulation, making it challenging to identify the date of ovulation. Nonetheless, HPR and TrendFilt both start the jump at day 16, correctly identifying the date of ovulation as day 15. While MedFilt places the jump correctly, it introduces excess motion in the pre- and post-ovulatory portions of the cycle. GPR and Adspline over-smooth, placing the beginning of temperature increase around day 12.

**FIGURE 5:**
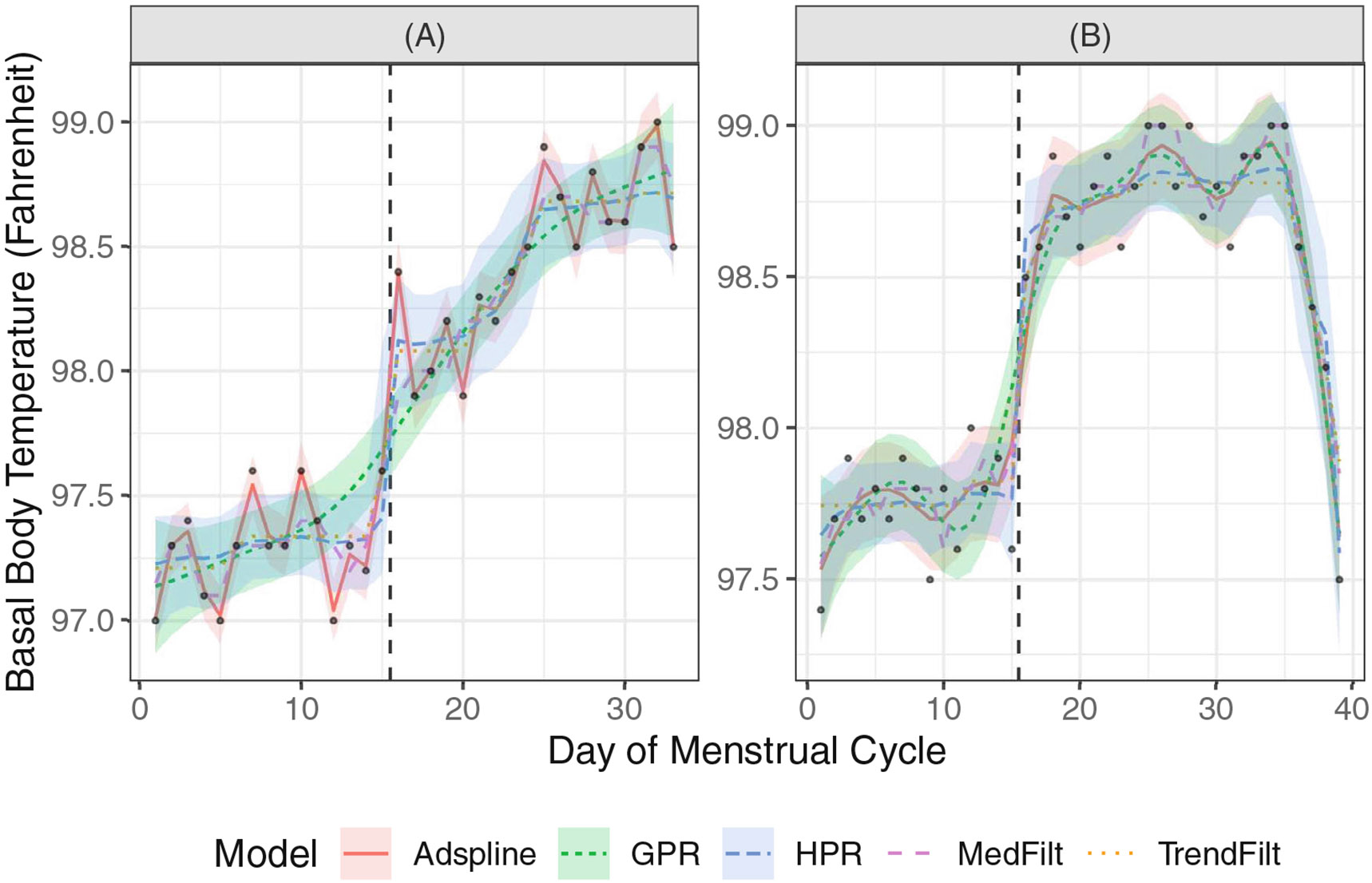
Fitted basal body temperature trajectory and 95% credible/confidence intervals from a horseshoe process regression (HPR), adaptive spline model (Adspline), Gaussian process regression (GPR), median filter (MedFilt), and penalized trend filter (TrendFilt) for two women who conceived a pregnancy. Observed datapoints are given as black dots, with an expert’s guess of the ovulation time given by the vertical black dashed line. Note that there is no information sharing across women; models are refit separately for each woman’s BBT trajectory. A color version of this figure is available online.

In Figure 5, we present two plots for women who conceived that cycle. In panel A, we see a successful pregnancy which features a second temperature shift at implantation. HPR and TrendFilt identify ovulation at day 15, and then somewhat capture the slightly higher BBT post-implantation, starting around day 23. GPR would completely smooth over the BBT trajectory for the pregnancy cycle, missing the dates of ovulation and implantation entirely, while Adspline and MedFilt overfit, making the fit difficult to interpret. In panel B, we show the plot of a pregnancy that ended in a miscarriage. HPR identifies the ovulation jump at day 15. The sustained high temperatures (lasting almost 20 days) indicate conception and implantation of an embryo. However, the dropping temperatures from day 36 are an early warning sign, resulting in a miscarriage on day 39.

**FIGURE 6:**
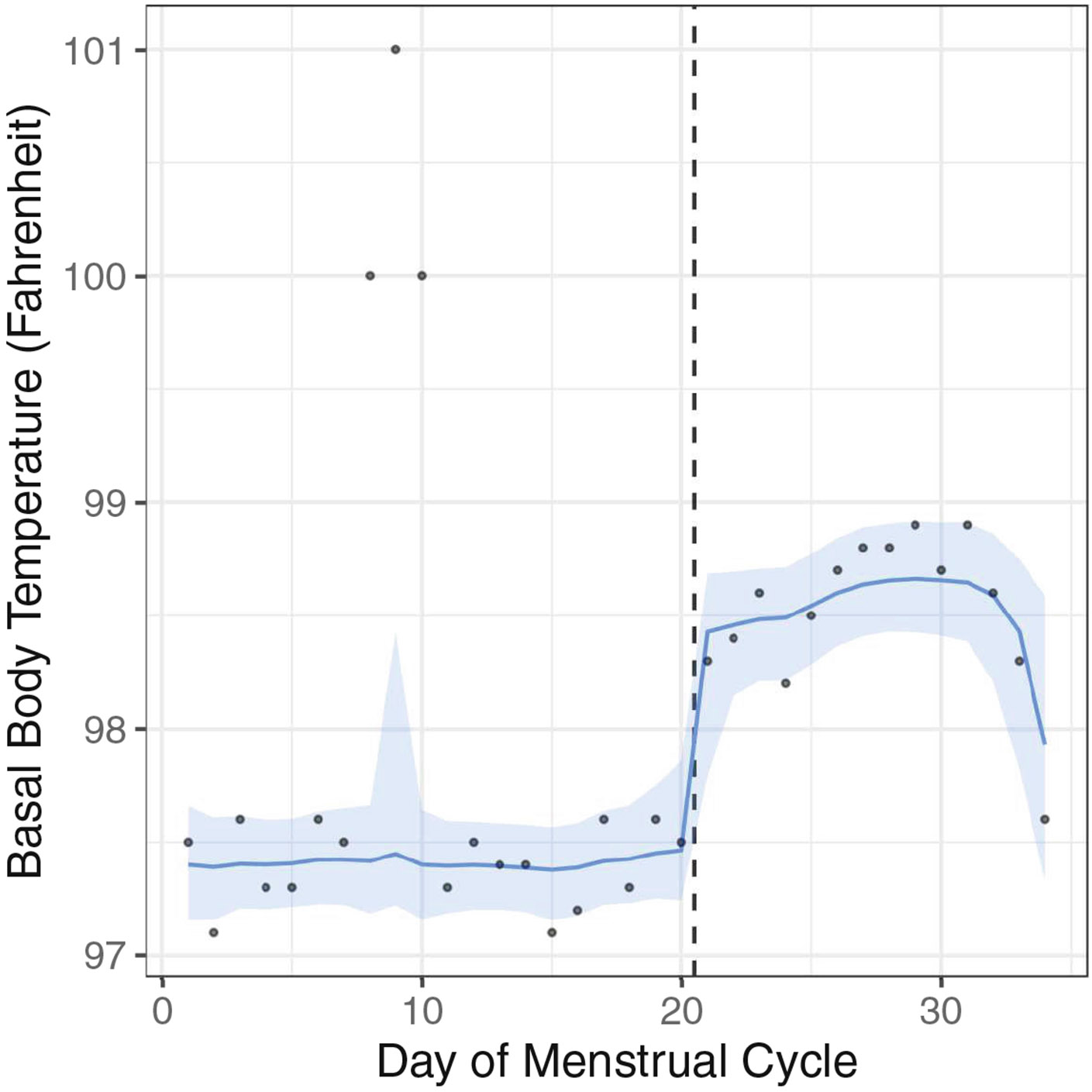
Fitted basal body temperature trajectory and 95% credible/confidence intervals from a horseshoe process regression (HPR) adjusted for the presence of fever for a woman who was ill during days 8–10 of her menstrual cycle. Observed datapoints are given as black dots, with an expert’s guess of the ovulation time given by the vertical black dashed line.

Finally, we give an example of the use of covariates in HPR. We restrict our focus to HPR because the other comparison methods either cannot include linear covariates (MedFilt, TrendFilt) or do not return easily interpretable nonlinear components in the presence of linear covariates (Adspline, GPR). This woman was sick from days 8–10 of her menstrual cycle, with a high fever. By including illness as an additional categorical linear covariate in the HPR, we can adjust away its effect. That results in the estimated BBT trajectory given in Figure 6, in which ovulation occurs on day 21, with the illness estimated to increase BBT by 2.88 (2.44, 3.27) degrees Fahrenheit.

## 6 ∣ DISCUSSION

We present HPR, a method for fitting functions that feature local changes in variability. HPR contributes to a growing literature on the use of local-global shrinkage families within a stochastic process framework.^10,11,19^ Here, we focus on the details of implementation, filling in some of the gaps of an overarching theoretical framework that has not been fully translated to applied use. We find that HPR outperforms other existing methods for fitting step functions and other associations with abrupt changes, although we would not recommend using it to fit an association that is expected to be smooth. Other methods like Gaussian process regression would likely yield better results. We extend HPR to allow for additional linear covariates, data interpolation, and monotonicity constraints. We find that our data interpolation scheme yields good results and that HPR’s superior performance for fitting step functions persists in the presence of additional linear covariates, even when correlated with the nonlinear predictor. Together, these extensions make HPR more usable for applied research on nonlinear associations with local changes in variance in small samples. HPR is available as an R package on GitHub.

Computational burden is an unresolved challenge in our implementation of HPR. We use Stan and cmdstanr, which yields good performance in our context and gives reasonable computing times and decent convergence diagnostics in a range of data-generating scenarios. However, computing time becomes prohibitive at larger sample sizes and is a limitation. Future work may wish to consider a Gibbs sampling type approach like that of Kowal et al^11^ or other approaches for scalable Bayesian inference like variational Bayes.^34^ Even with extensive tuning and debugging, we were unable to fully eliminate HMC divergences from model implementation, a problem that seems common to the horseshoe prior.^10,17^ We see no evidence that these sporadic HMC divergences affect performance, although it is something of which to be aware. In general, if other convergence diagnostics (effective samples, Gelman-Rubin convergence diagnostic, max treedepth warnings) are acceptable, it seems mostly safe to ignore sporadic divergences occuring in < 5% of samples. If HMC divergences are more frequent than that or combined with other evidence of nonconvergence, we would recommend further tuning of the model or the use of a different method. Future work may also wish to examine whether the regularized horseshoe prior within HPR resolves some of these issues without sacrificing model performance.^17^

Our data interpolation scheme motivated new questions about the theory and implementation of horseshoe process prior models. To date, all implementations of horseshoe process prior models have chosen to use a discrete formulation, as we do here.^10,11^ Although this simplifies mathematical derivation and computation, it requires imputation or approximations to conduct data interpolation and augmentation. We chose to use a Bayesian imputation scheme. In an ideal world, it would be possible to develop a horseshoe process in a truly continuous formulation and leverage properties of the multivariate Meixner distribution to calculate predictions and interpolations without needing to rerun the MCMC. However, current mathematical theory does not make that possible. We attempted several *ad hoc*, stop-gap measures that allowed us to impute new predictions without rerunning the MCMC, such as imputing the local shrinkage parameter for the new predictor as the mean of its two nearest local shrinkage parameter neighbors, and then using the imputed local shrinkage parameter within Gaussian kriging equations to generate a prediction.^23^ We did not have success with these approximations and thus chose the more statistically rigorous Bayesian imputation approach we outline here. Future work may wish to investigate this and other approximations for horseshoe process prediction and kriging further.

We have demonstrated the utility of HPR and shown that it is usable for a variety of real-world data. We find that HPR is an excellent choice for data featuring local changes in variance, such as step functions or piecewise linear functions. Future work will further elucidate HPR’s strengths and weaknesses and provide new insights into computationally efficient and stable implementations and its underlying theory.

## Supplementary Material

Supplement

Additional supporting information can be found online in the Supporting Information section at the end of this article.

## Data Availability

All code and data needed to reproduce this analysis can be found at https://github.com/elizabethchase/HPR and https://github.com/elizabethchase/HPR_Sims.
