# Supplementary material for: Modeling basal body temperature data using horseshoe process regression
Source: Stat Med. Author manuscript; Available in PMC 2026 Feb 24. (PMC12930000; doi:10.1002/sim.9991)
Supplement: Supplement [file NIHMS2139642-supplement-Supplement.pdf]

Supporting Information for "Modeling Basal Body Temperature Data Using Horseshoe Process Regression" by Elizabeth C. Chase, Jeremy M. G. Taylor, Philip S. Boonstra

---

Contents

1 Simulation Results: Binary and Count Data 2

2 Simulation Results: Pointwise Performance 7

3 Different Types of Monotonicity Constraints 11

4 Augmentation Results: Binary and Count Outcomes 15

5 Partial Linear Models 18

6 Computational Assessment 27

7 Effects of Hyperparameters and Sample Size 32

1 Simulation Results: Binary and Count Data

While the main manuscript focused on continuous outcomes, here we explore the performance of HPR for binary and count outcomes via simulation. We considered four true underlying associations, each of which were observed at an equally spaced grid of n = 100 observations for count data and n = 150 observations for binary data:

1. bigstep: $f(x) = 0 * I(x \leq 2) + 6 * I(2 < x \leq 5) + 1 * I(5 < x \leq 6) + 3 * I(6 < x \leq 8) + 10 * I(x > 8)$ (divided by 10 for binary data).

2. bounce: $f(x) = |sin(x)|$ (multiplied by 10 for count data).

3. impulse: $f(x) = 0*I(x = 0)+\exp(-x)*I(0 < x < 3)+1*I(x = 3)+\exp(-(x-3))*I(3 < x < 7) + \exp(-(x-7))*I(x = 7)$ (multiplied by 5 for count data).

4. joinpoint: $f(x) = (1.5x) * I(x < 2) + (16 - 5x) * I(2 \leq x < 3) + 1 * I(3 \leq x < 6) + (10 - x) * I(6 \leq x < 9) + (5x - 44) * (x \geq 9)$ (divided by 6 for binary data).

$I()$ denotes the indicator function; i.e. $I(x) = 1$ if condition x is true, and $I(x) = 0$ otherwise. We compared horseshoe process regression (HPR) to Gaussian process regression (GPR) and adaptive splines (Adspline). Unlike in the main manuscript, we did not consider the median filter (MedFilt) or trend filter (TrendFilt) because these methods are not implemented for noncontinuous outcomes.

We assessed performance with three primary metrics:

1. Mean absolute difference (MAD): $\frac{1}{n}\sum_{i=1}^{n} |g^{-1}(f(x_i)) - g^{-1}(\hat{f}(x_i))|$, where $g^{-1}(\hat{f}(x_i))$ is the predicted function's value at x_i on the mean scale and $g^{-1}(f(x_i))$ is the true function's value at x_i on the mean scale.

2. Credible/confidence interval width (Width): $\frac{1}{n}\sum_{i=1}^{n} g^{-1}(\hat{f}(x_i)^{0.975}) - g^{-1}(\hat{f}(x_i)^{0.025})$, where $\hat{f}(x_i)^{0.975}$ denotes the upper bound of a 95% credible/confidence interval for $\hat{f}(x_i)$ and $\hat{f}(x_i)^{0.025}$ is the lower bound. Both bounds are transformed back to the mean scale using

g^{-1} to assess credible interval width.

3. Credible/confidence interval coverage (Coverage): $\frac{1}{n}\sum_{i=1}^{n} I(g^{-1}(\hat{f}(x_i)^{0.025}) \leq g^{-1}(f(x_i)) \leq g^{-1}(\hat{f}(x_i)^{0.975}))$.

We assessed performance on each metric across the 100 replicates of each of our 3 data-generating scenarios for each method. All code used to completely reproduce the simulations can be found on GitHub.

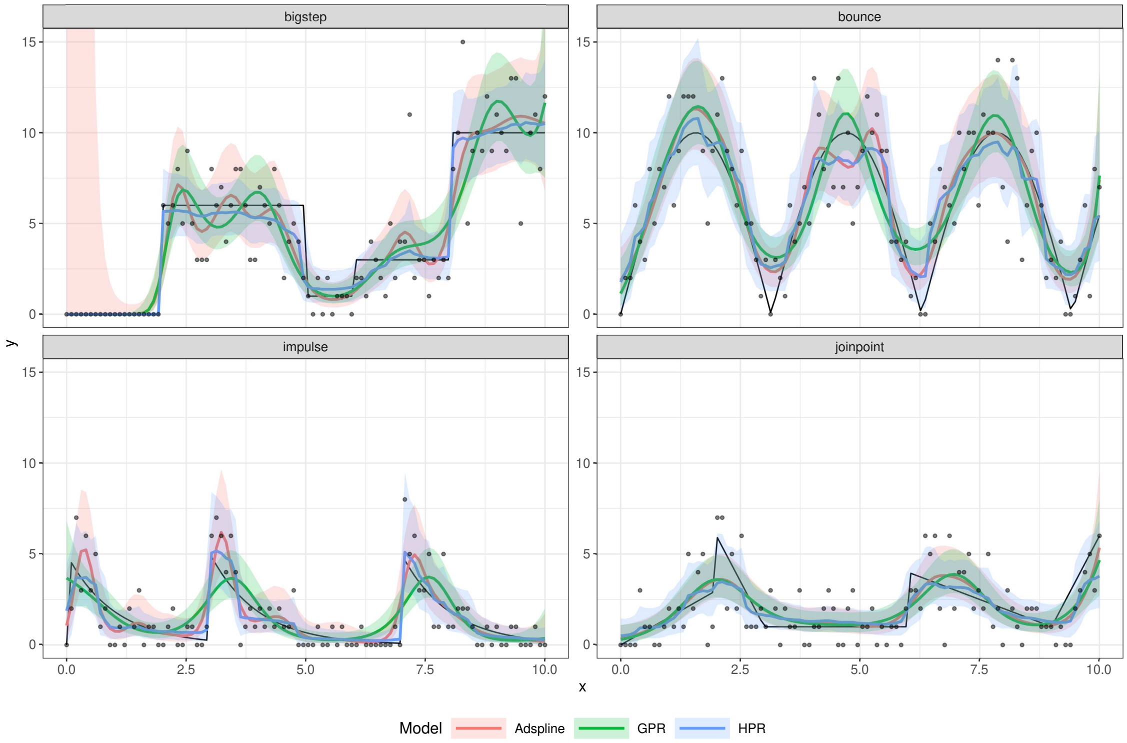

Figure S1: *Point estimates and 95% credible/confidence intervals for horseshoe process regression (HPR), adaptive splines (Adspline), and Gaussian process regression (GPR) for count data. Each sample dataset has n = 100.*

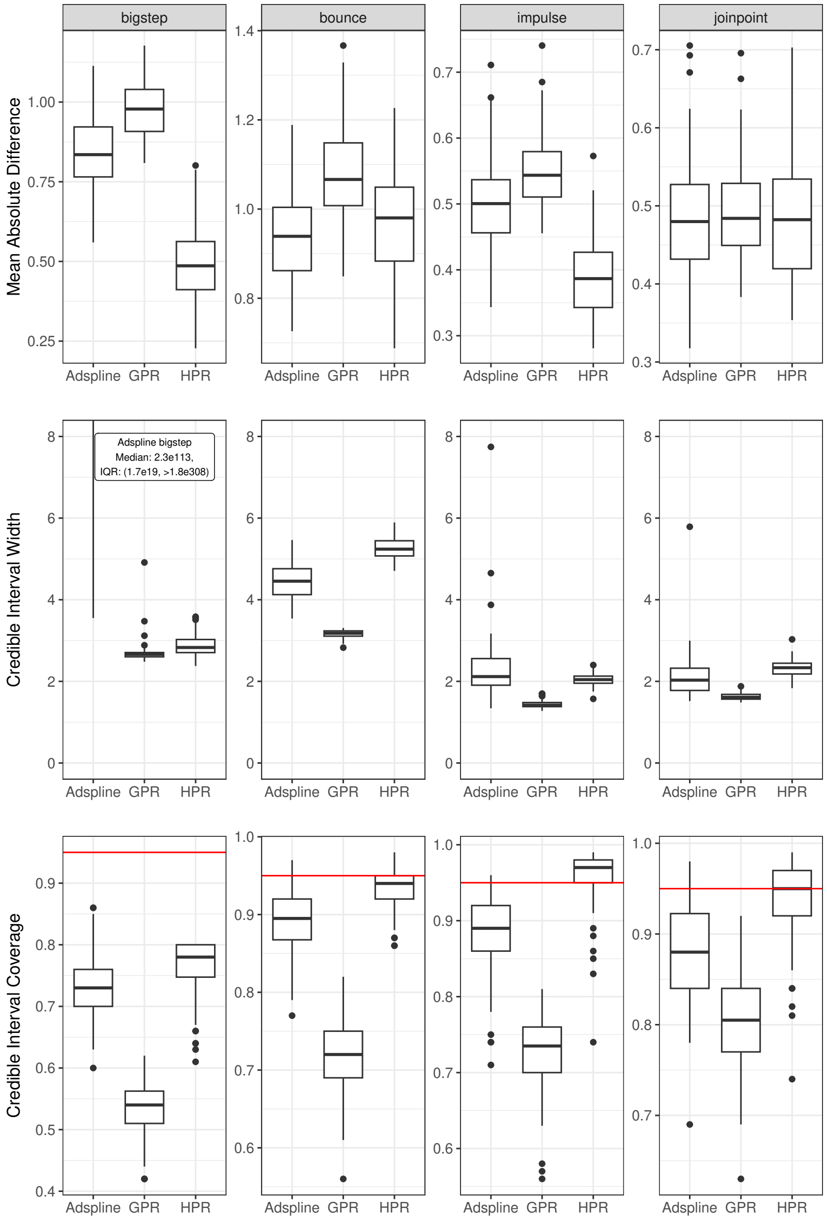

Figure S2: *Horseshoe process regression (HPR) simulation results for count data, based on 100 replicates on three data-generating scenarios, each with $n = 100$. Comparison methods were adaptive splines (Adspline) and Gaussian process regression (GPR). The top row gives performance for mean absolute difference (smaller is better); the second row gives performance for credible/confidence interval width; the third row gives performance for credible/confidence interval coverage (0.95 is nominal and given as a red line). Each column is for one data-generating scenario; sample datasets are given in S1.*

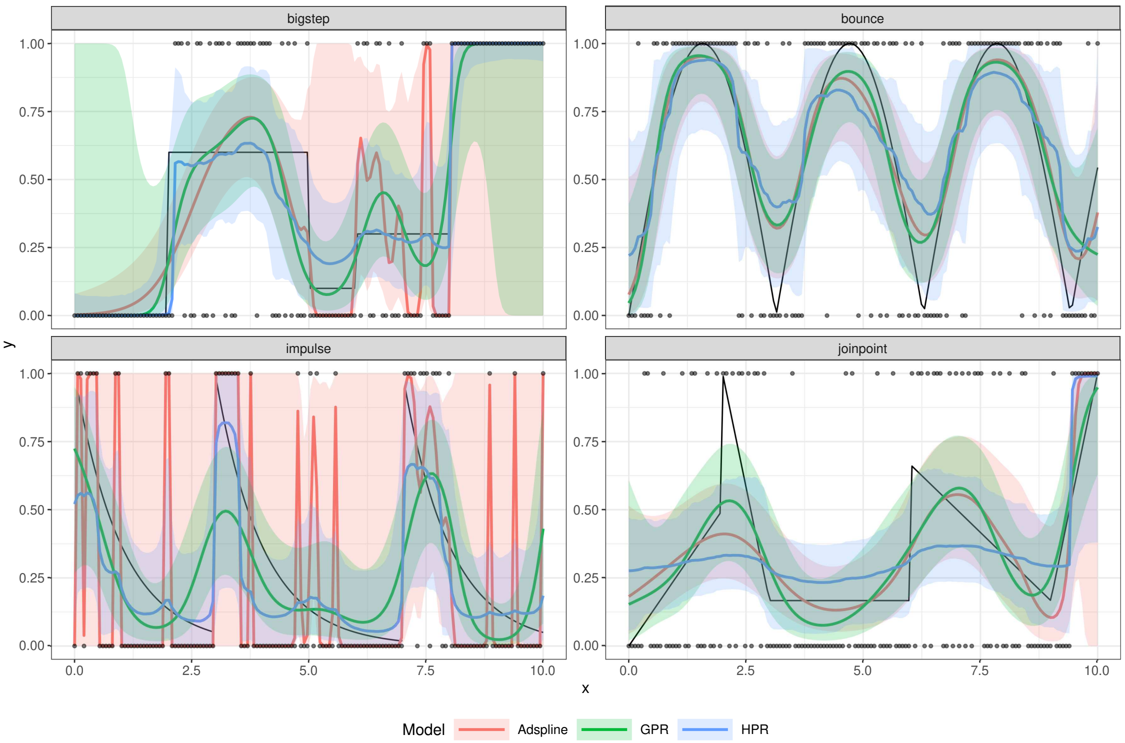

Figure S3: *Point estimates and 95% credible/confidence intervals for horseshoe process regression (HPR), adaptive splines (Adspline), and Gaussian process regression (GPR) for binary outcomes. Each sample dataset has* $n = 150$*.*

Several findings are worthy of further note. First, the adaptive spline model (Adspline) had a great deal of trouble for count outcomes in the bigstep scenario, with extremely large credible intervals and highly erratic fits (Figures S1 and S2). It does not seem suited to this setting. Gaussian process regression (GPR) and horseshoe process regression (HPR) returned more sensible fits, with HPR excelling in the bigstep scenario. However, all methods struggled to maintain nominal credible interval coverage in the bigstep scenario; HPR was closest to nominal of the comparison methods (Figures S2 and S4). For binary outcomes, there was evidence that HPR was overshrinking, particularly in the impulse and joinpoint scenarios (Figure S3). This might be improved with a higher value of c, the scale on the prior of τ.

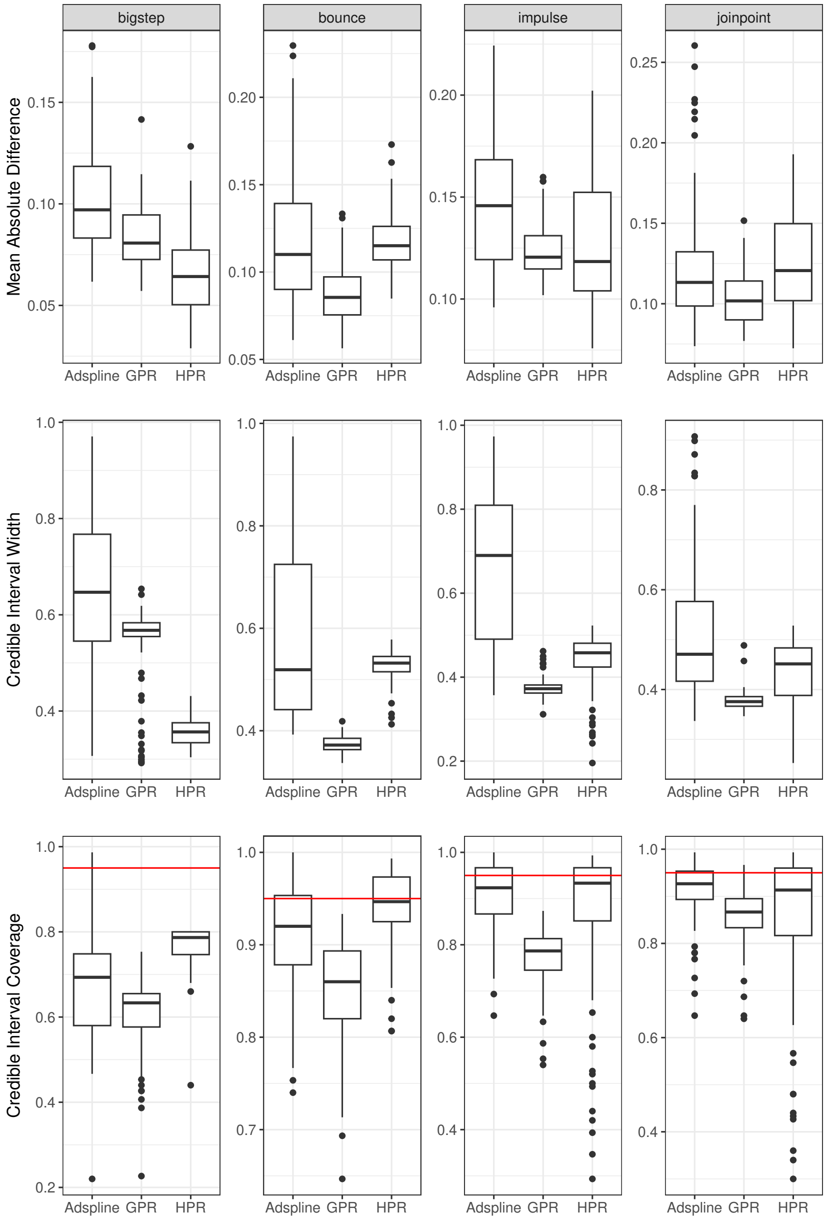

Figure S4: *Horseshoe process regression (HPR) simulation results for binary outcomes, based on 100 replicates on four data-generating scenarios, each with n = 150. Comparison methods were Gaussian process regression (GPR) and adaptive splines (Adspline). The top row gives performance for mean absolute difference (smaller is better); the second row gives performance for credible/confidence interval width; the third row gives performance for credible/confidence interval coverage (0.95 is nominal and given as a red line). Each column is for one data-generating scenario; sample datasets are given in S3.*

2 Simulation Results: Pointwise Performance

In addition to the aggregate outcomes presented in the main manuscript and in the section above, we also considered pointwise performance (not summed across all observed datapoints):

1. Pointwise bias: $f(x_i) - \hat{f}(x_i)$.
2. Pointwise credible/confidence interval width: $\hat{f}(x_i)^{0.975} - \hat{f}(x_i)^{0.025}$.
3. Pointwise credible/confidence interval coverage: $I(\hat{f}(x_i)^{0.025} \leq f(x_i) \leq \hat{f}(x_i)^{0.975})$.

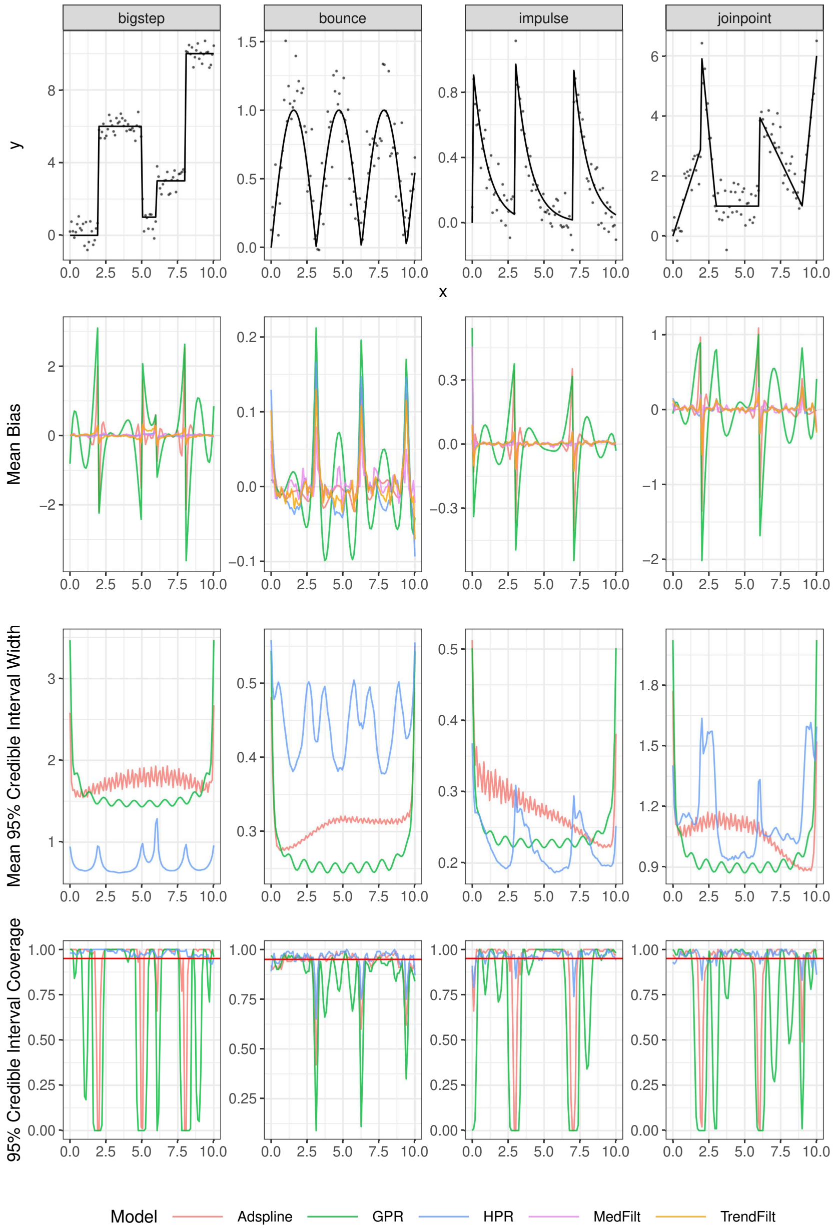

Figure S5: *Pointwise simulation results for continuous outcomes, based on 100 replicates on four data-generating scenarios, each with n = 100. Comparison methods were adaptive splines (Adspline), Gaussian process regression (GPR), median filter (MedFilt), and the penalized trend filter (TrendFilt). The top row gives a sample dataset and the true trajectory. The second row gives performance for mean bias, averaged over the 100 replicates at each point (smaller is better); the third row gives credible interval width, averaged over the 100 replicates at each point; the fourth row gives performance for credible interval coverage at each point (0.95 is nominal and given as a red line). Each column is for one data-generating scenario.*

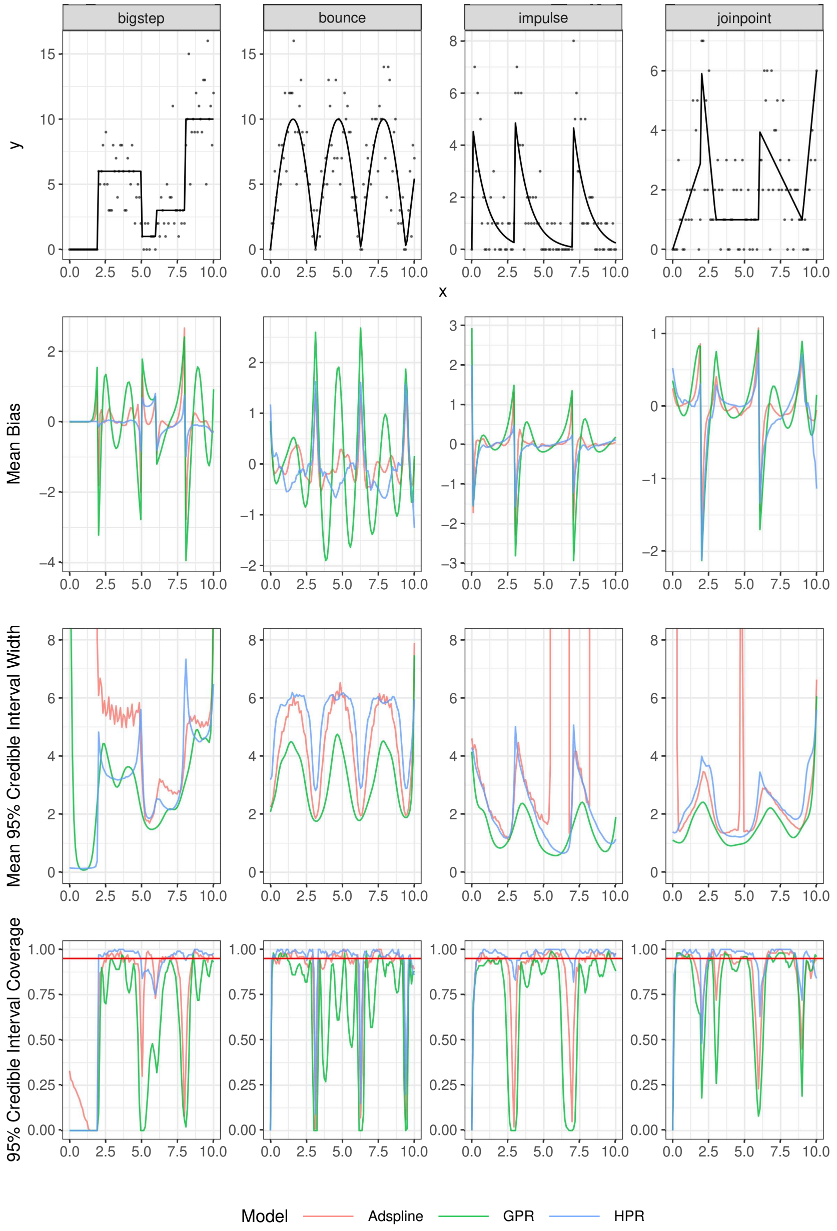

Figure S6: *Pointwise simulation results for count data, based on 100 replicates on four data-generating scenarios, each with n = 100. Comparison methods were adaptive splines (Adspline) and Gaussian process regression (GPR). The top row gives a sample dataset and the true trajectory. The second row gives performance for mean bias, averaged over the 100 replicates at each point (smaller is better); the third row gives credible interval width, averaged over the 100 replicates at each point; the fourth row gives performance for credible interval coverage at each point (0.95 is nominal and given as a red line). Each column is for one data-generating scenario.*

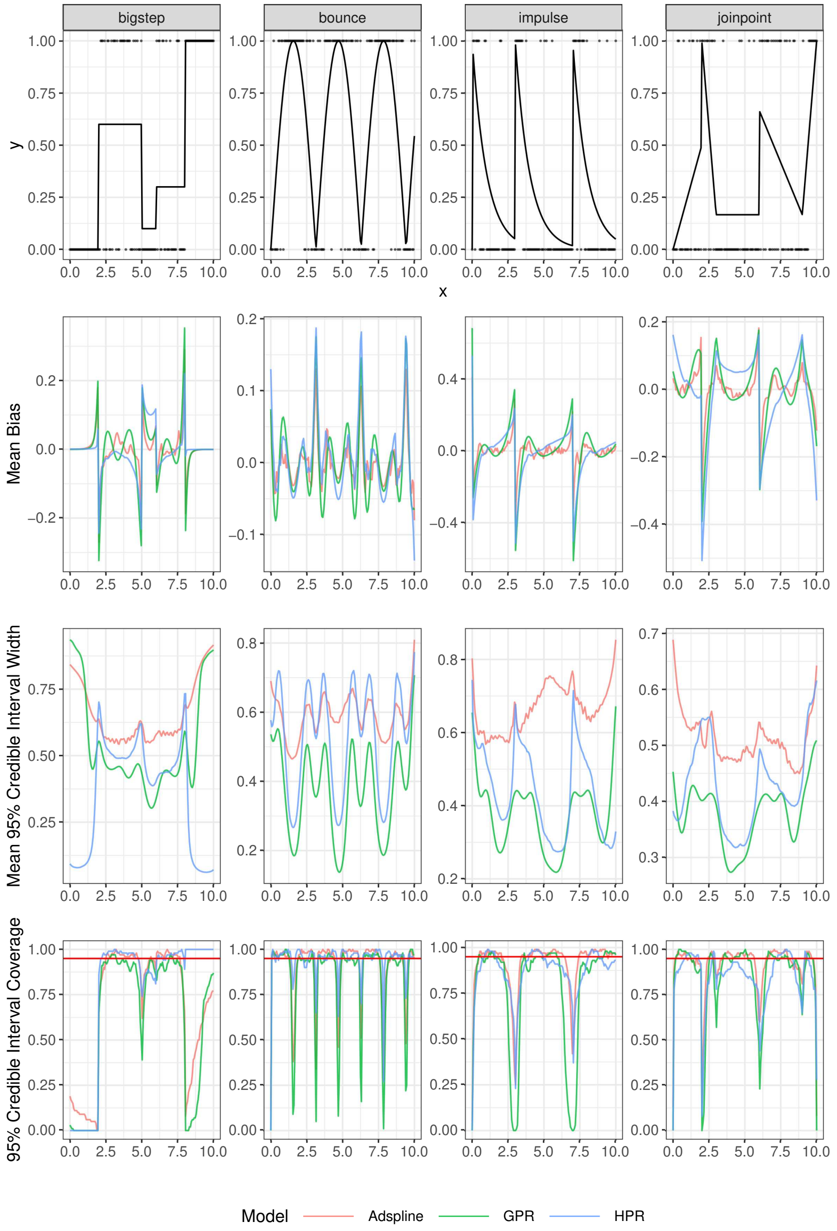

Figure S7: *Pointwise simulation results for binary outcomes, based on 100 replicates on four data-generating scenarios, each with $n = 100$. Comparison methods were adaptive splines (Adspline) and Gaussian process regression (GPR). The top row gives a sample dataset and the true trajectory. The second row gives performance for mean bias, averaged over the 100 replicates at each point (smaller is better); the third row gives credible interval width, averaged over the 100 replicates at each point; the fourth row gives performance for credible interval coverage at each point (0.95 is nominal and given as a red line). Each column is for one data-generating scenario.*

Pointwise performance was generally similar to aggregate performance. All methods showed the worst performance at the location of the abrupt jumps, with increased mean absolute difference and decreased coverage. Although HPR also showed this worsened performance, it did better at capturing the jumps than the other comparison methods. In the case of binary outcomes, HPR was likely to return flat-line fits, again suggesting that a larger value of c, the prior scale on τ, may be advisable for binary outcomes (Figure S7).

3 Different Types of Monotonicity Constraints

We proposed using the absolute value function to constrain the association between x and y to be monotonic. To do so, we modify our HPR to be:

$$g(E(y_i)) = f_j = \alpha + \sum_{k=1}^{j} |h_k| \tag{1}$$

Although we chose to use the absolute value, note that many other functions could be used to impose a monotonicity constraint–any function that transforms from the reals to the positive reals would be able to achieve this goal. We also considered using the exp function in lieu of the absolute value function. We present simulation results here to justify the choice of absolute value function.

We generated 100 evenly spaced predictors values between 0 and 10. Then, we considered two monotonic data-generating scenarios:

1. bigstep: $f(x) = 0 * I(x \leq 2) + 6 * I(2 < x \leq 5) + 10 * I(5 < x \leq 6) + 12 * I(6 < x \leq 8) + 20 * I(x > 8)$.

2. smooth: $f(x) = \log(\frac{x/11+0.01}{1-(x/11+0.01)})$.

We simulated Gaussian noise around each true curve with standard deviation of 1 for the bigstep scenario and standard deviation of 0.5 for the smooth scenario. Then, we compared

the unconstrained HPR to an HPR constrained using the absolute value function (HPR_abs) and an HPR constrained using exponentiation (HPR_exp). We considered both estimation performance and computational metrics.

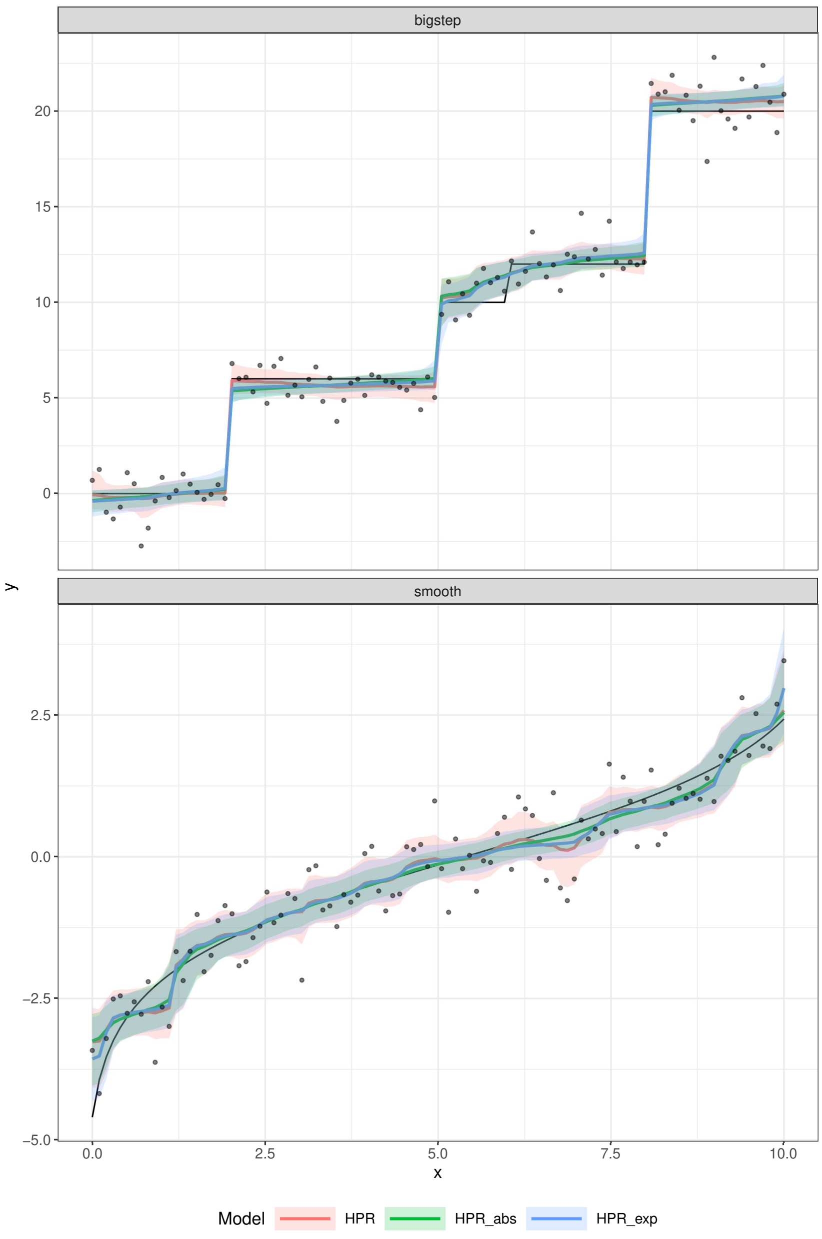

Figure S8: *Point estimates and 95% credible/confidence intervals for an HPR with no constraint (HPR), a constrained HPR via absolute value (HPR_abs), and a constrained HPR via exponentiation (HPR_exp) for continuous outcomes. Each sample dataset has $n = 100$.*

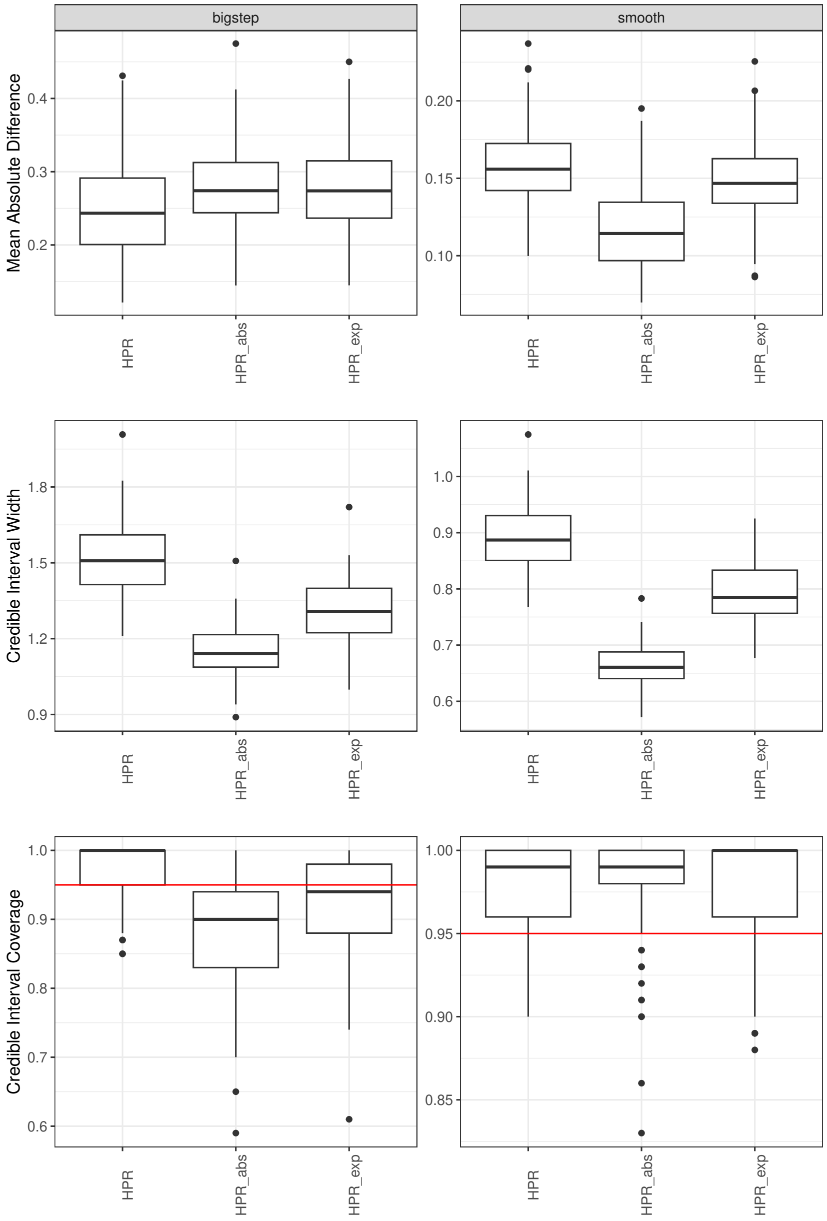

Figure S9: *Simulation results for a horseshoe process regression (HPR) constrained to be monotonic increasing, based on 100 replicates on two data-generating scenarios, each with n = 100. Comparison methods were an HPR with no constraint (HPR), a constrained HPR via absolute value (HPR_abs), and a constrained HPR via exponentiation (HPR_exp). The top row gives performance for mean absolute difference (smaller is better); the second row gives performance for credible interval width; the third row gives performance for credible interval coverage (0.95 is nominal and given as a red line). Each column is for one data-generating scenario; sample datasets can be seen in Figure S8.*

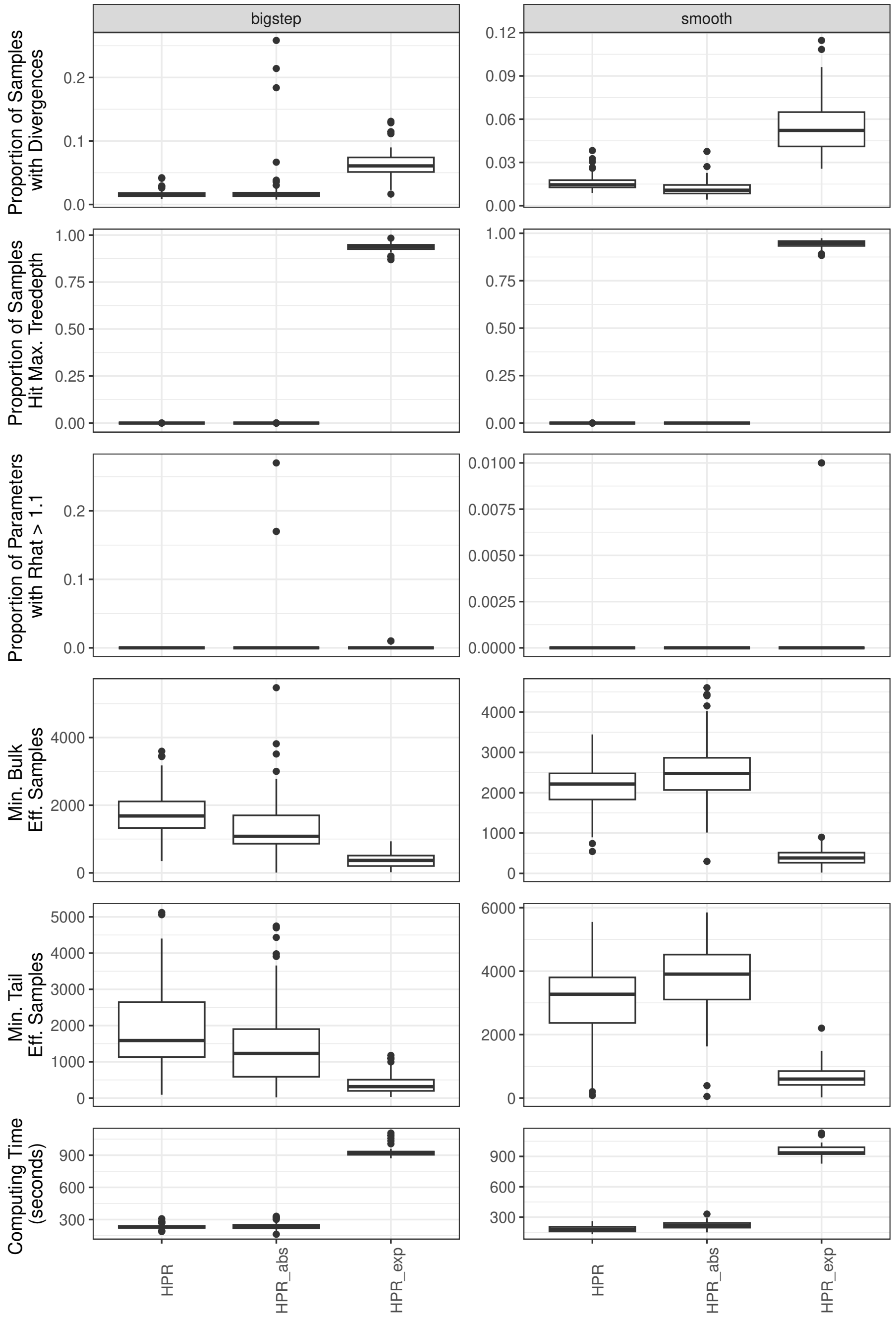

Figure S10: *Computational performance of a horseshoe process regression (HPR) constrained to be monotonic increasing using either no constraint (HPR), absolute value (HPR_abs), or exponentiation (HPR_exp) based on 100 replicates in two data-generating scenarios for Gaussian outcomes. Smaller is better for all metrics except Min. Bulk. Eff. Samples and Min. Tail. Eff. Samples (the minimum effective sample size in the bulk and tails of the posterior, respectively). Each column is for one data-generating scenario.*

Results are given in Figures S8, S9, and S10. From these, we see that estimation performance of the absolute-value constrained HPR was superior in terms of mean absolute difference and credible interval width, although coverage was slightly lower than nominal for the bigstep scenario. However, the computational performance is the real justification for using the absolute value transformation–the exponentiation constraint returned a substantially higher proportion of divergences, max tree-depth warnings, and other nonconvergence signals–in addition to being substantially slower.

4 Augmentation Results: Binary and Count Outcomes

Here, we explored the performance of our data augmentation scheme for binary and count outcomes. We only considered scenarios bigstep and bounce (described above in Section 1). We randomly sampled 100 unevenly spaced datapoints between 0 and 10 to be our observed x locations (150 datapoints for binary data). Then, we fit the HPR either 1) only using the observed datapoints, 2) augmented by a grid of datapoints at every 0.5 (roughly 20 augmented datapoints), and 3) augmented by a grid of datapoints at every 0.1 (roughly 100 augmented datapoints). We calculated the performance metrics described above separately for the observed datapoints and the augmented datapoints, to see if predictions at the observed datapoints changed depending on the number of gridpoints, and if predictions at the augmented datapoints were fairly accurate.

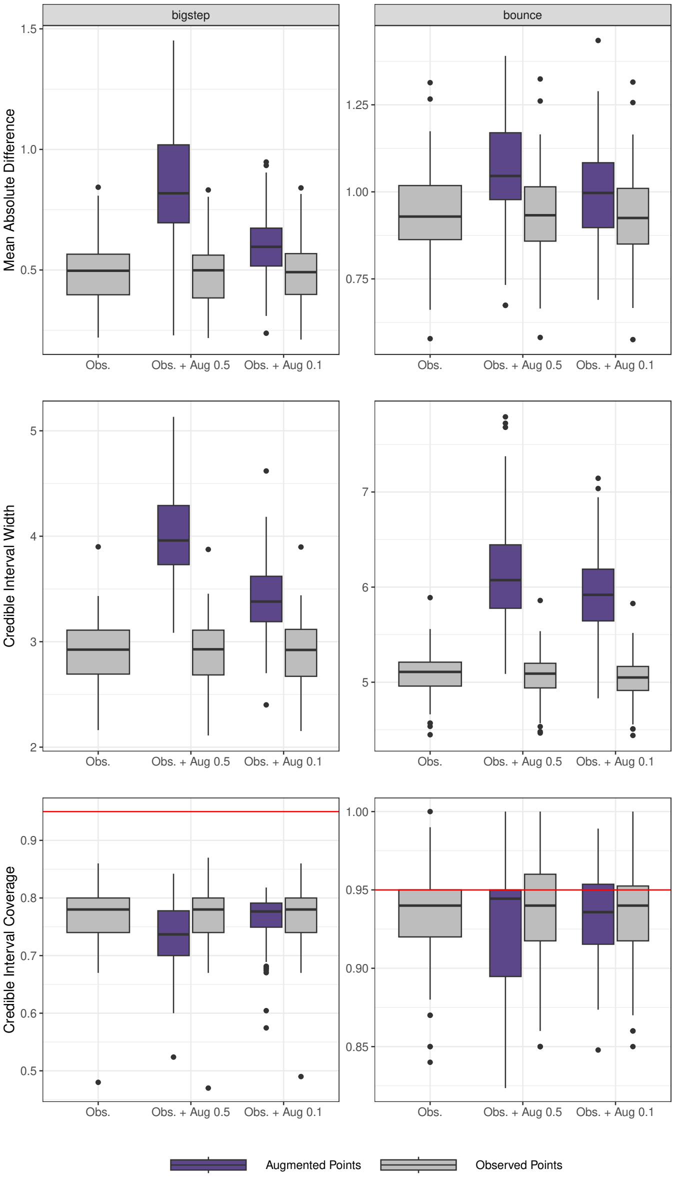

Figure S11: *Horseshoe process regression (HPR) data augmentation simulation results for count outcomes, based on 100 replicates on two data-generating scenarios. We compared an HPR calculated only at $n = 100$ observed points (Obs) to an HPR with augmentation points at a grid of every 0.5 (Obs + Aug 0.5) and an HPR with augmentation points at a grid of every 0.1 (Obs + Aug 0.1) from 0 to 10. The top row gives performance for mean absolute difference calculated at both the observed and augmented points (smaller is better); the second row gives performance for credible interval width calculated at both the observed and augmented points; the third row gives credible interval coverage calculated at both the observed and augmented points (0.95 is nominal and marked as a red line). Performance at observed points and augmented points are displayed separately. Each column is for one data-generating scenario.*

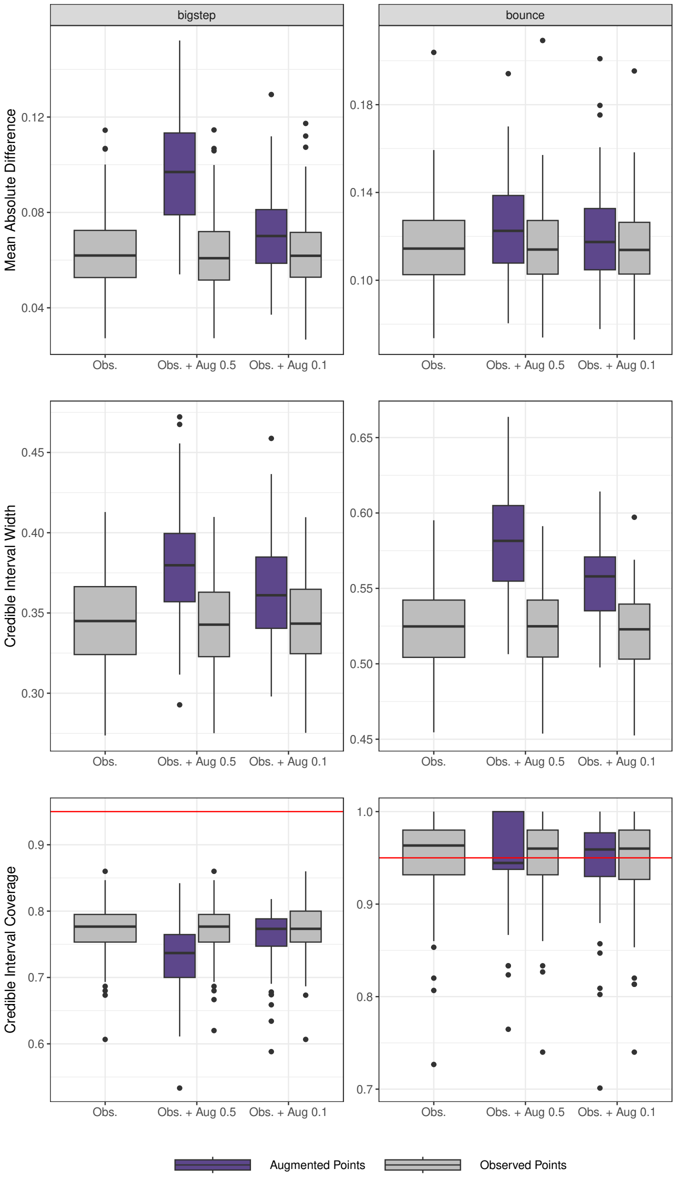

Figure S12: *Horseshoe process regression (HPR) data augmentation simulation results for binary outcomes, based on 100 replicates on two data-generating scenarios. We compared an HPR calculated only at n = 100 observed points (Obs) to an HPR with augmentation points at a grid of every 0.5 (Obs + Aug 0.5) and an HPR with augmentation points at a grid of every 0.1 (Obs + Aug 0.1) from 0 to 10. The top row gives performance for mean absolute difference calculated at both the observed and augmented points (smaller is better); the second row gives performance for credible interval width calculated at both the observed and augmented points; the third row gives credible interval coverage calculated at both the observed and augmented points (0.95 is nominal and marked as a red line). Performance at observed points and augmented points are displayed separately. Each column is for one data-generating scenario.*

Results of the augmentation scheme for binary and count outcomes largely resembled the performance for continuous outcomes presented in the main manuscript. Performance at observed datapoints did not change with varying numbers of augmentation datapoints. However, aggregate performance at augmentation datapoints improved with increased grid density, with reduced mean absolute difference and narrower credible intervals. This "improved performance" is somewhat misleading, because in the data generating schemes considered here–which do not feature an extremely large number of abrupt changes–the augmentation scheme will do better with more augmentation points as a matter of probability. With more augmentation points, the probability that an augmentation point is placed at the location of an abrupt jump is reduced, artificially boosting aggregate performance. In general, the model fit estimated at an augmentation point was an interpolation of the two nearest observed datapoints, with credible intervals that reflected the increased uncertainty.

5 Partial Linear Models

We assessed the performance of our HPR partial linear model. We simulated five covariates. The first three of these covariates were simulated from a multivariate normal distribution with mean vector $(67, 0, 130)$ and standard deviation vector $(7, 1, 20)$ with correlation of 0.35:

$$\begin{pmatrix} X_1 \\ X_2^* \\ X_3 \end{pmatrix} \sim MVN\left(\begin{pmatrix} 67 \\ 0 \\ 130 \end{pmatrix}, \begin{pmatrix} 7^2 & 7*1*0.35 & 7*20*0.35 \\ 7*1*0.35 & 1^2 & 1*20*0.35 \\ 7*20*0.35 & 1*20*0.35 & 20^2 \end{pmatrix}\right)$$

The second of these covariates was made into a binary variable by splitting it at 24: $X_2 = I(X_2^* > 24)$.

The remaining 2 covariates were simulated independently from a multivariate normal distribution with mean vector $(80, 45)$, standard deviation vector $(20, 12)$, and correlation of 0.2:

$$\begin{pmatrix} X_4 \\ X_5 \end{pmatrix} \sim MVN\left(\begin{pmatrix} 80 \\ 45 \end{pmatrix}, \begin{pmatrix} 20^2 & 20 * 12 * 0.2 \\ 20 * 12 * 0.2 & 12^2 \end{pmatrix}\right)$$

For continuous outcomes, the first 4 of these covariates were assumed to have a linear relationship with the outcome y, with coefficient vector $(\beta_1, \beta_2, \beta_3, \beta_4) = (0, 5, 0.05, 0.1)$. We considered two different functional forms for the fifth covariate:

1. bigstep: $f(x_5) = 0 * I(x_5 < 35) + 5 * I(35 \leq x_5 < 55) + 6 * I(55 \leq x_5 < 65) + 8 * I(65 \leq x_5 < 80) + 10 * I(x_5 \geq 80)$

2. smooth: $f(x_5) = \frac{x_5^2}{100}$

Thus $E(y) = 0 * X_1 + 5 * X_2 + 0.05 * X_3 + 0.1 * X_4 + f(X_5)$. We then generated observations for 100 subjects, and simulated Gaussian error with $\sigma = 0.5$.

For count data, the first 4 of these covariates were assumed to have a linear relationship with the outcome $\log(E(y))$, with coefficient vector $(\beta_1, \beta_2, \beta_3, \beta_4) = (0, 0.08, 0.01, 0.01)$. We considered two different functional forms for the fifth covariate:

1. bigstep: $f(x_5) = 0 * I(x_5 < 35) + 0.5 * I(35 \leq x_5 < 55) + 0.8 * I(55 \leq x_5 < 65) + 1 * I(65 \leq x_5 < 80) + 1.3 * I(x_5 \geq 80)$

2. smooth: $f(x_5) = |sin(x_5)|$

Thus $\log(E(y)) = 0 * X_1 + 0.08 * X_2 + 0.01 * X_3 + 0.01 * X_4 + f(X_5)$. We simulated 100 datapoints as described above, calculated $\log(E(y))$, transformed to the $E(y)$ scale, and randomly sampled 100 observed outcomes from a Poisson distribution.

For binary data, we used coefficient vector $(\beta_1, \beta_2, \beta_3, \beta_4) = (0, -0.5, -0.05, 0.1)$ for the linear relationship with outcome $\log(E(y)/(1 - E(y)))$. We considered two different functional forms for the fifth covariate:

1. bigstep: $f(x_5) = -5 * I(x_5 < 35) + 0 * I(35 \leq x_5 < 55) + 1 * I(55 \leq x_5 < 65) + 5 * I(65 \leq x_5 < 80) + 10 * I(x_5 \geq 80)$

2. smooth: $f(x_5) = sin(x_5)$

Thus $\log(E(y)/(1 - E(y))) = 0 * X_1 - 0.5 * X_2 - 0.05 * X_3 + 0.1 * X_4 + f(X_5)$. We simulated 150 datapoints as described above, calculated $\log(E(y)/(1 - E(y)))$, transformed to the $E(y)$ scale, and randomly sampled 150 observed outcomes from a Bernoulli distribution.

We compared HPR to GPR and Adspline. We did not consider the median filter (MedFilt) or trend filter (TrendFilt) because these methods are not implemented for additional linear covariates. We assessed performance using the mean absolute difference between true $E(y)$ and estimated $\hat{E}(y)$, averaged over all datapoints, and width and coverage of the 95% credible intervals of $E(y)$ averaged over all datapoints. We also considered bias and coverage for the coefficients for the linear predictors.

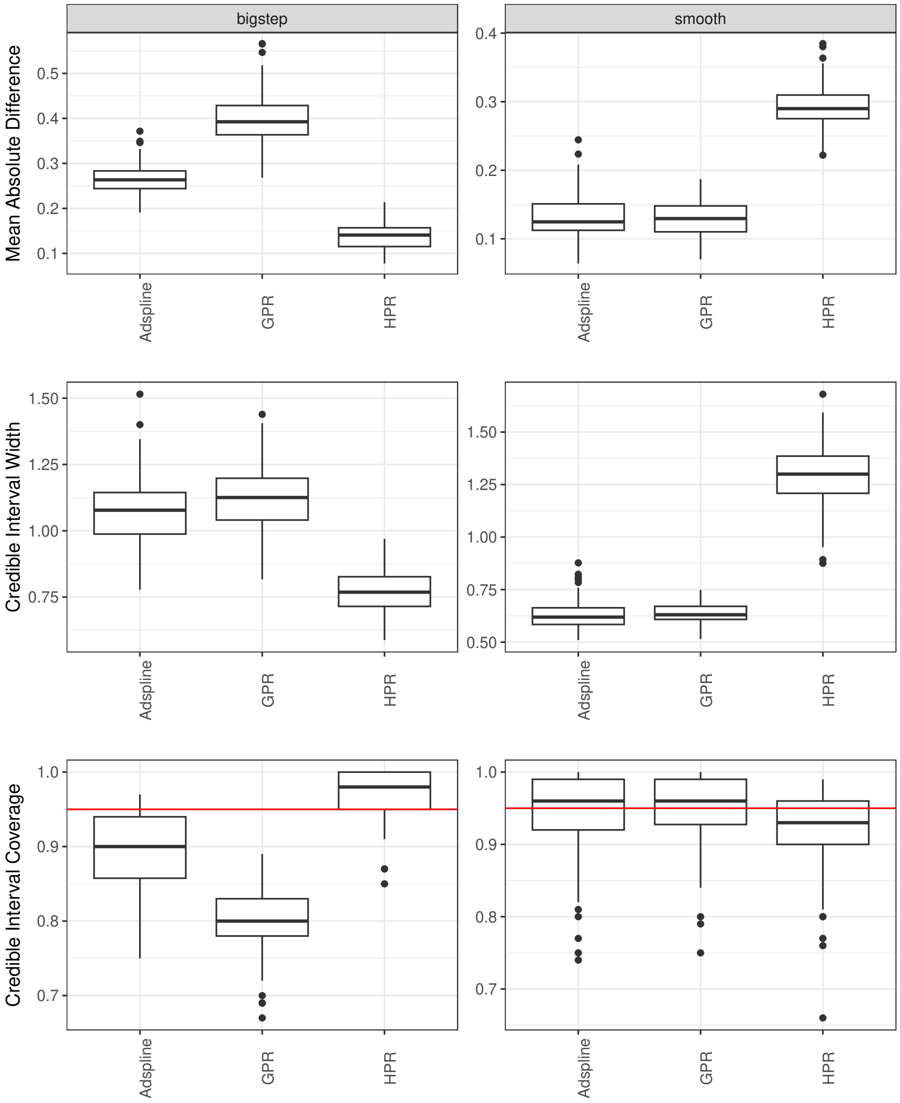

Figure S13: *Performance of a horseshoe process regression (HPR) partial linear model for estimating continuous outcomes, based on 100 replicates on two data-generating scenarios with* $n = 100$*. Comparison methods were Gaussian process regression (GPR) and adaptive splines (Adspline). The top row gives performance for mean absolute difference between the true outcome* $E(y)$ *and estimated outcome* $\hat{E}(y)$ *(smaller is better); the second row gives 95% credible interval width around* $\hat{E}(y)$*; the third row gives performance for credible interval coverage of* $E(y)$ *(0.95 is nominal and marked as a red line). Each column is for one data-generating scenario.*

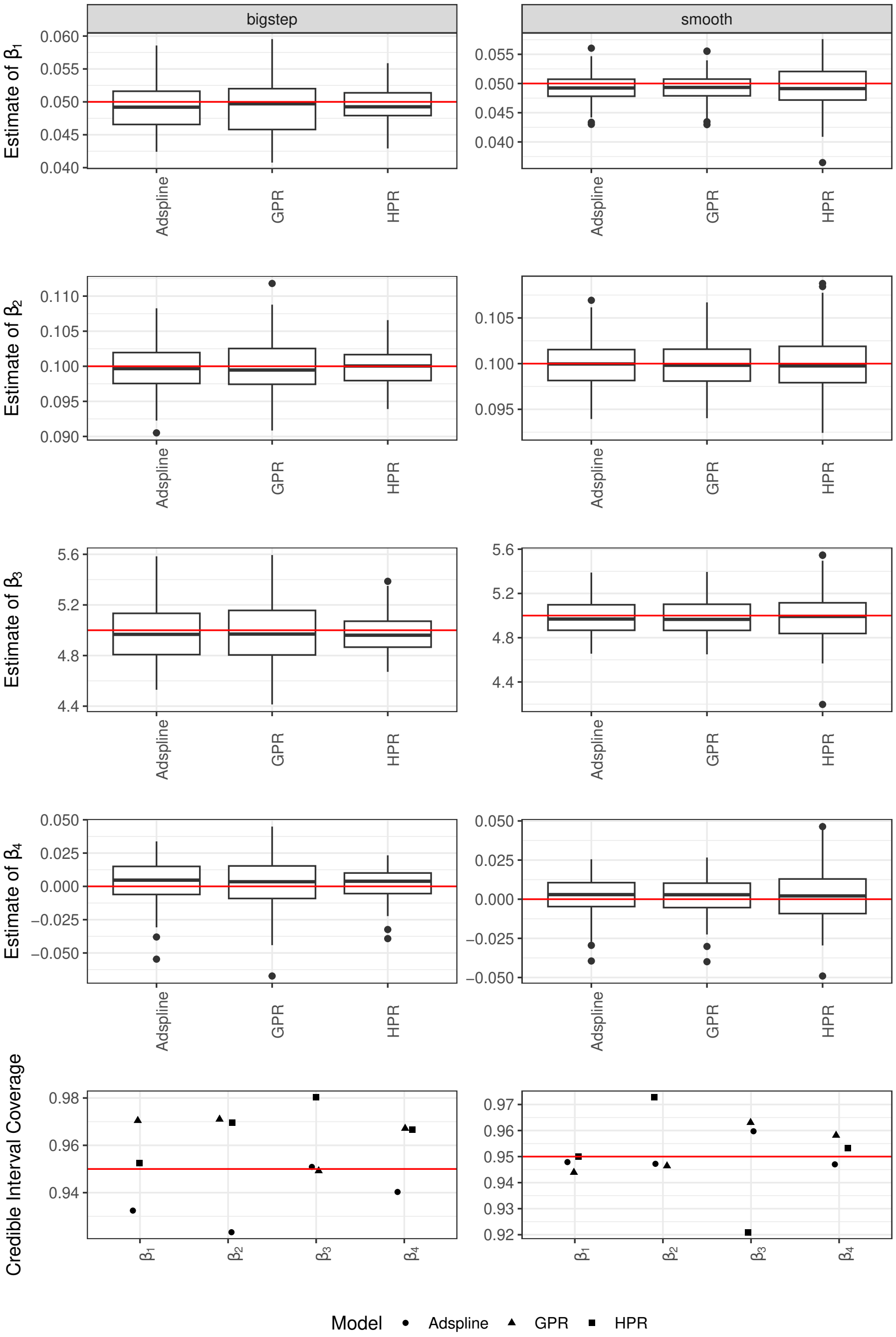

Figure S14: *Performance of a horseshoe process regression (HPR) Gaussian partial linear model for fitting four linear predictors, based on 100 replicates of two data-generating scenarios for the nonlinear predictor with $n = 100$. Comparison methods were Gaussian process regression (GPR) and adaptive splines (Adspline). The first four rows give the estimates of each of the coefficients, with the correct value given as a red line; the fifth row gives performance for credible interval coverage for all four coefficients (0.95 is nominal and marked as a red line). Each column is for one data-generating scenario.*

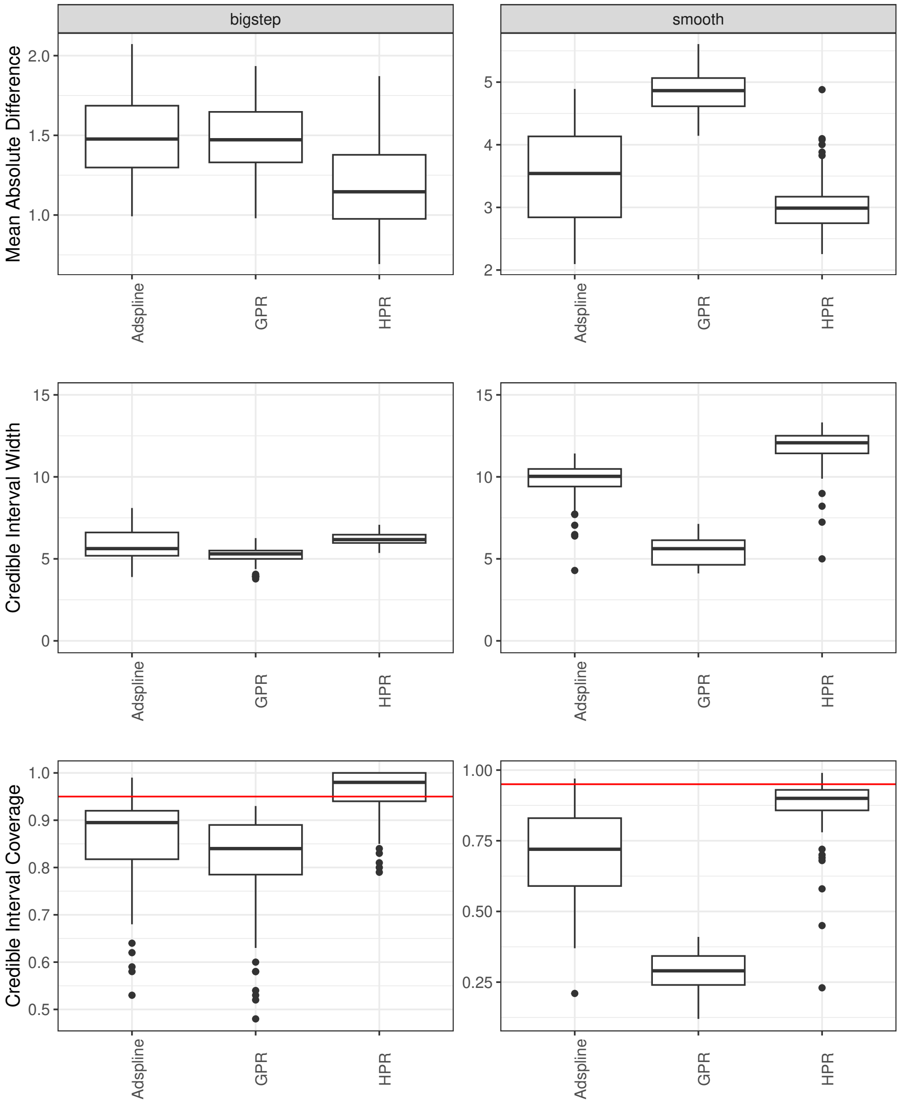

Figure S15: *Performance of a horseshoe process regression (HPR) partial linear model for estimating count outcomes, based on 100 replicates on two data-generating scenarios with n = 100. Comparison methods were Gaussian process regression (GPR) and adaptive splines (Adspline). The top row gives performance for mean absolute difference between the true outcome* $E(y_i)$ *and estimated outcome* $\hat{E}(y_i)$ *(smaller is better) averaged over 100 datapoints; the second row gives performance for credible interval width averaged over 100 datapoints; the third row gives coverage of* $E(y_i)$*, averaged over 100 datapoints (0.95 is nominal and marked as a red line). Each column is for one data-generating scenario. Note that the y-axis for credible interval width is truncated due to one extremely wide credible interval for HPR in the bigstep scenario.*

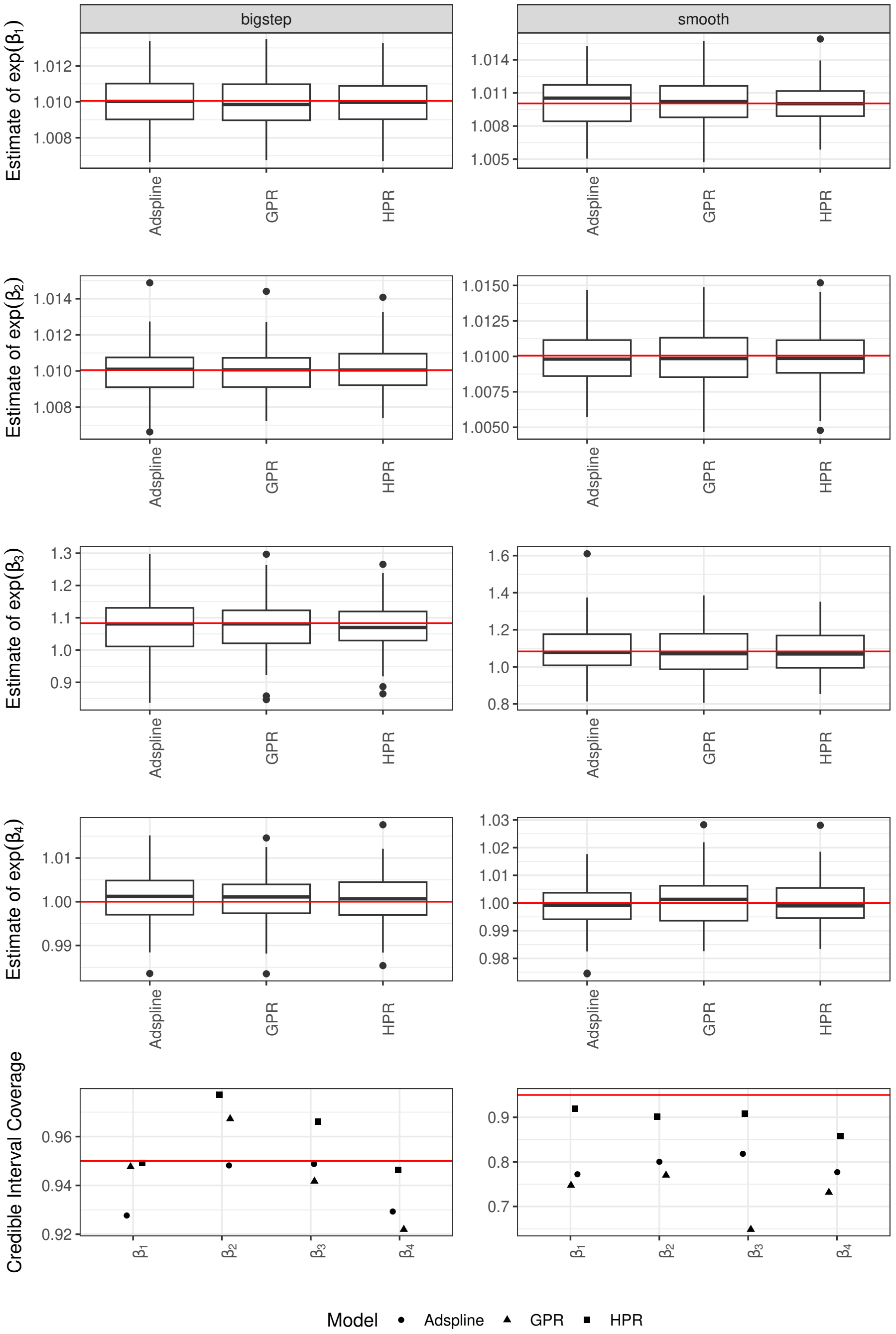

Figure S16: *Performance of a horseshoe process regression (HPR) Poisson partial linear model for fitting four linear predictors, based on 100 replicates on two data-generating scenarios for the nonlinear predictor with $n = 100$. Comparison methods were Gaussian process regression (GPR) and adaptive splines (Adspline). The first four rows give the estimates of each of the exponentiated coefficients, with the correct value given as a red line; the fifth row gives performance for credible interval coverage for all four exponentiated linear predictors (0.95 is nominal and marked as a red line). Each column is for one data-generating scenario.*

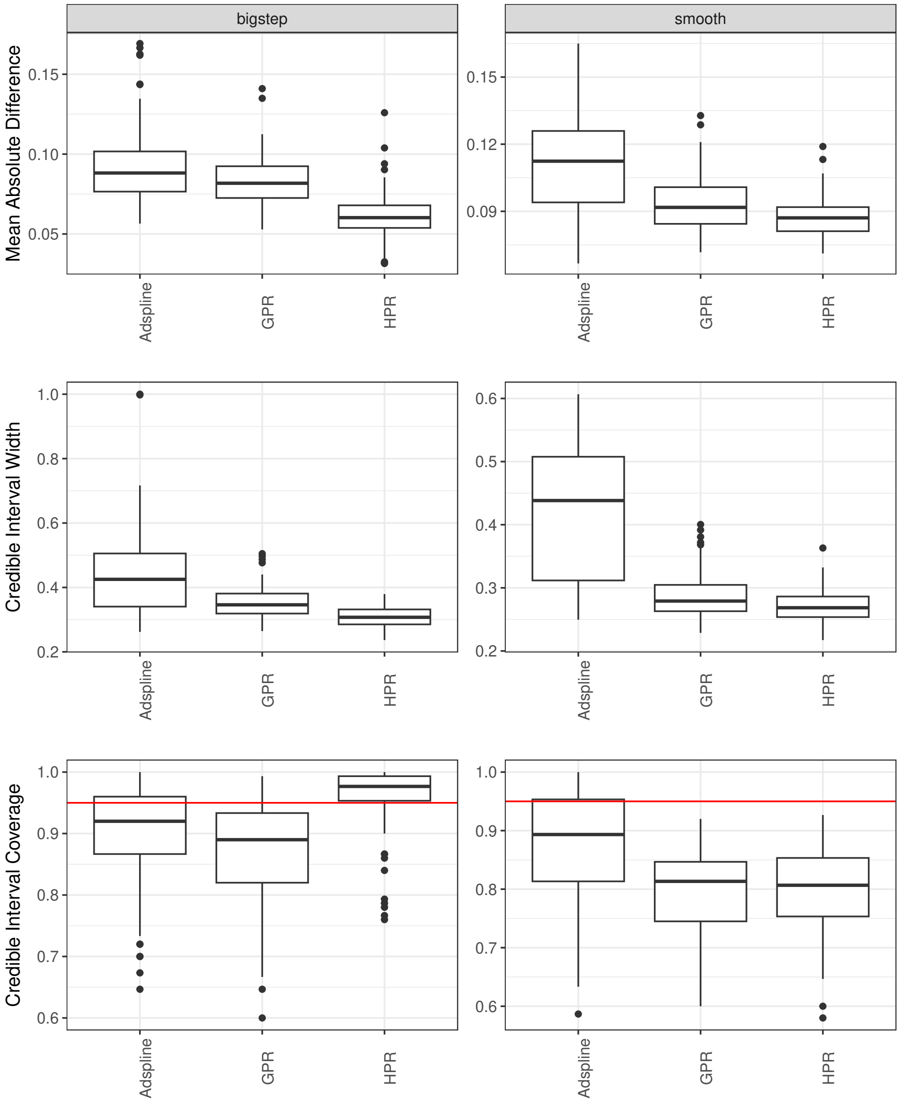

Figure S17: *Performance of a horseshoe process regression (HPR) partial linear model for estimating binary outcomes, based on 100 replicates on two data-generating scenarios with n = 150. Comparison methods were Gaussian process regression (GPR) and adaptive splines (Adspline). The top row gives performance for mean absolute difference between the true outcome* $E(y_i)$ *and estimated outcome* $\hat{E}(y_i)$ *(smaller is better) averaged over 150 datapoints; the second row gives performance for credible interval width averaged over the 150 datapoints; the third row gives coverage of* $E(y_i)$*, averaged over the 150 datapoints (0.95 is nominal and marked as a red line). Each column is for one data-generating scenario.*

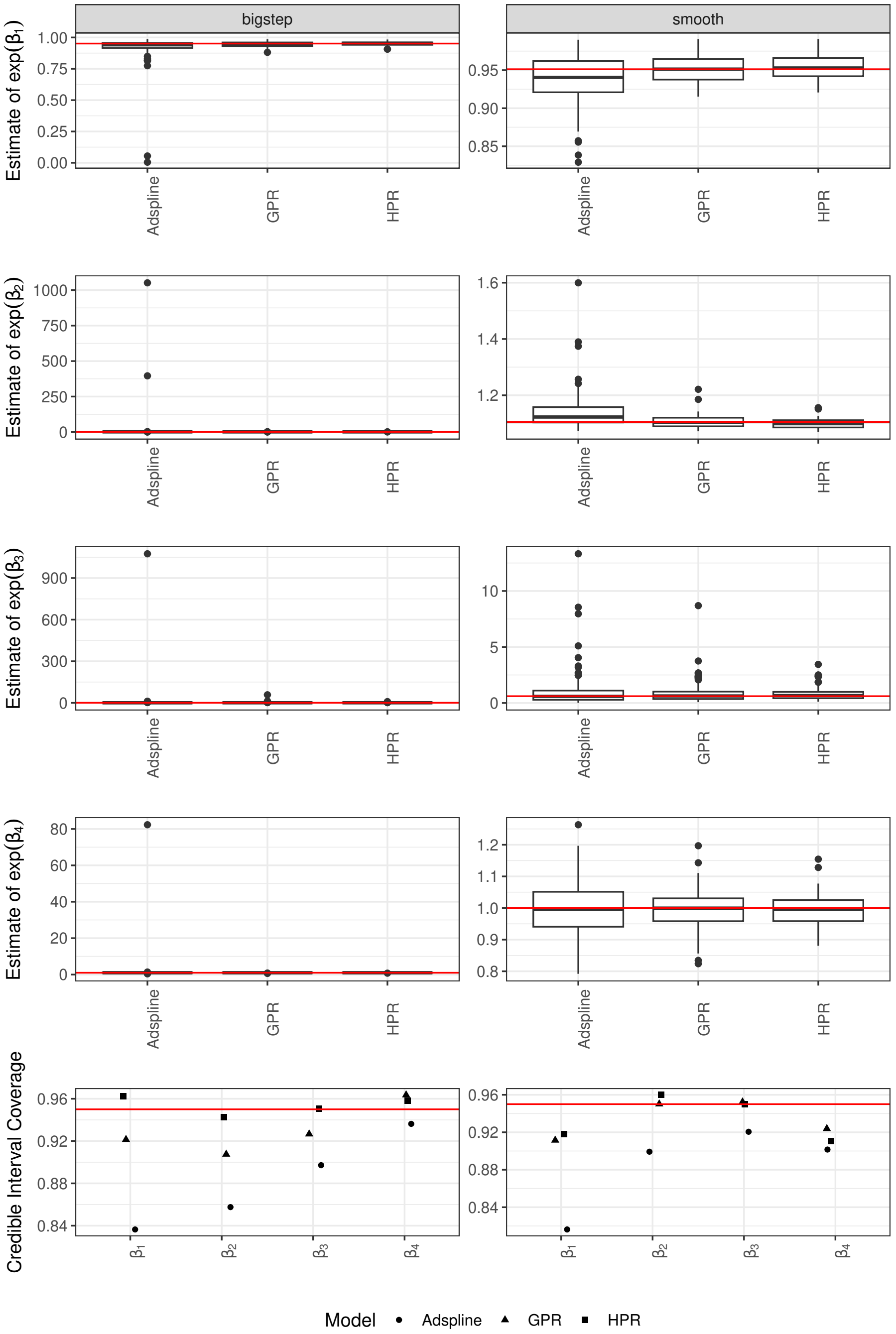

Figure S18: *Performance of a horseshoe process regression (HPR) Bernoulli partial linear model for fitting four linear predictors, based on 100 replicates on two data-generating scenarios for the nonlinear predictor with $n = 150$. Comparison methods were Gaussian process regression (GPR) and adaptive splines (Adspline). The first four rows give the estimates of each of the exponentiated coefficients, with the correct value given as a red line; the fifth row gives performance for credible interval coverage for all four exponentiated linear predictors (0.95 is nominal and marked as a red line). Each column is for one data-generating scenario.*

Performance of the partial linear model was generally good. HPR offered substantially reduced mean absolute difference and credible interval width for the latent mean $\hat{E}(y_i)$ when $f(X_5)$ was a step function. When $f(X_5)$ was a smooth function, its performance was worse than the comparison methods for continuous outcomes (Figure S13), although credible interval coverage was still very good. For binary and count outcomes, HPR consistently surpassed the comparison methods, even when $f(X_5)$ was a smooth function (Figures S15 and S17). The Gaussian process regression (GPR) particularly struggled for count outcomes. Regardless of the form of $f(X_5)$, performance for estimating the linear effects $(\beta_1, \beta_2, \beta_3, \beta_4)$ was good (Figures S14, S16, S18).

6 Computational Assessment

When conducting Bayesian modeling, it is important to assess model convergence and computational performance. We considered 6 computational metrics:

1. Proportion of MCMC samples that ended in an HMC divergence: This diagnostic is unique to Bayesian models fit using HMC. A divergence suggests that posterior sampling for that MCMC sample went "off the rails" and may be unreliable. In general, even a single divergence is cause for concern; however, as we discuss, in the case of HPR we think that small numbers of divergences (<5%) may be unavoidable and do not negatively affect model performance. We would like this metric to be close to 0.

2. Proportion of MCMC samples that ended in a max treedepth warning: This diagnostic is also unique to Stan models and indicates whether the No-U-Turn-Sampler was frequently taking the maximum number of steps in Hamiltonian space without hitting a U-turn, suggesting that the step size was too small. In some cases this is indicative of model nonconvergence/poor posterior exploration. It often corresponds to slower computational times. We would like this metric to be close to 0.

3. Proportion of parameters with $\hat{R} > 1.1$: This diagnostic compares between- and within-chain estimates of each parameter of the model (all HPR models are fit with 4 chains). Larger values of $\hat{R}$ suggest that the chains have not mixed well and that posterior estimates may be unstable. Ideally $\hat{R} < 1.05$ or $\hat{R} < 1.1$; we used 1.1 here, and considered what proportion of the model's parameters have $\hat{R} > 1.1$. We used the $\hat{R}$ proposed by Vehtari et al. (2021). We would like this metric to be close to 0.

4. Minimum bulk effective sample size: This diagnostic uses rank-normalized draws to estimate the effective sample size in the bulk of the posterior, as described in Vehtari et al. (2021). It is calculated for each parameter; here, we present the minimum sample size across all parameters. We would like this metric to be large, and ideally larger than 400.

5. Minimum tail effective sample size: This diagnostic uses rank-normalized draws to estimate the effective sample size in the tails of the posterior, as described in Vehtari et al. (2021). It is calculated for each parameter; here, we present the minimum sample size across all parameters. We would like this metric to be large, and ideally larger than 400.

6. Computational time: This metric tells how long it took the model to fit, in seconds. We would like this metric to be small.

For more information on these diagnostics, please see the Stan reference manual.

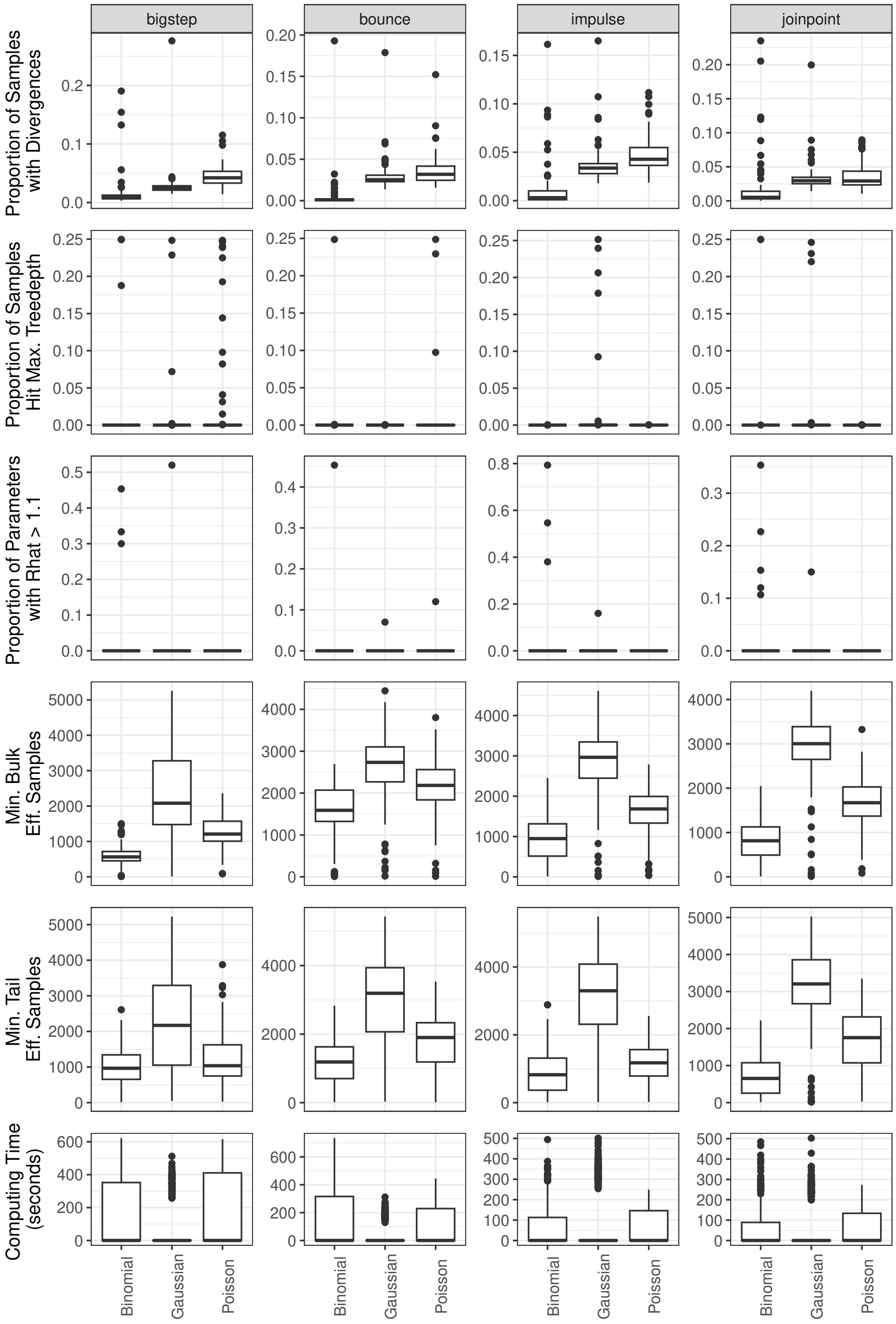

Figure S19: *Computational performance of a horseshoe process regression (HPR), based on 100 replicates in three data-generating scenarios, for continuous, binary, and count outcomes. Smaller is better for all metrics except Min. Bulk. Eff. Samples and Min. Tail. Eff. Samples (the minimum effective sample size in the bulk and tails of the posterior, respectively). Each column is for one data-generating scenario.*

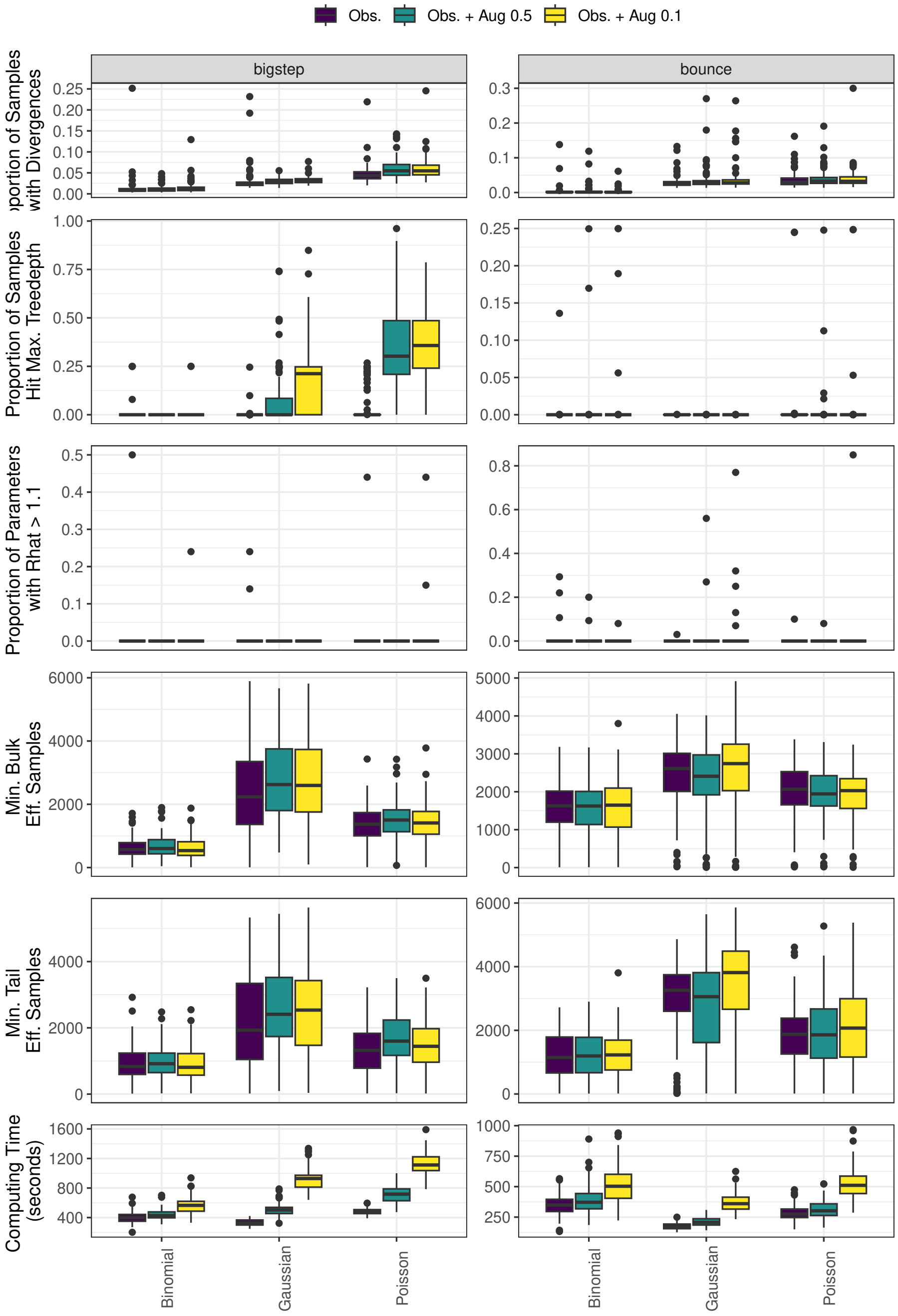

Figure S20: *Computational performance of a horseshoe process regression (HPR) in the presence of data interpolation, based on 100 replicates in three data-generating scenarios, for continuous, binary, and count outcomes. Smaller is better for all metrics except Min. Bulk. Eff. Samples and Min. Tail. Eff. Samples (the minimum effective sample size in the bulk and tails of the posterior, respectively). Each column is for one data-generating scenario.*

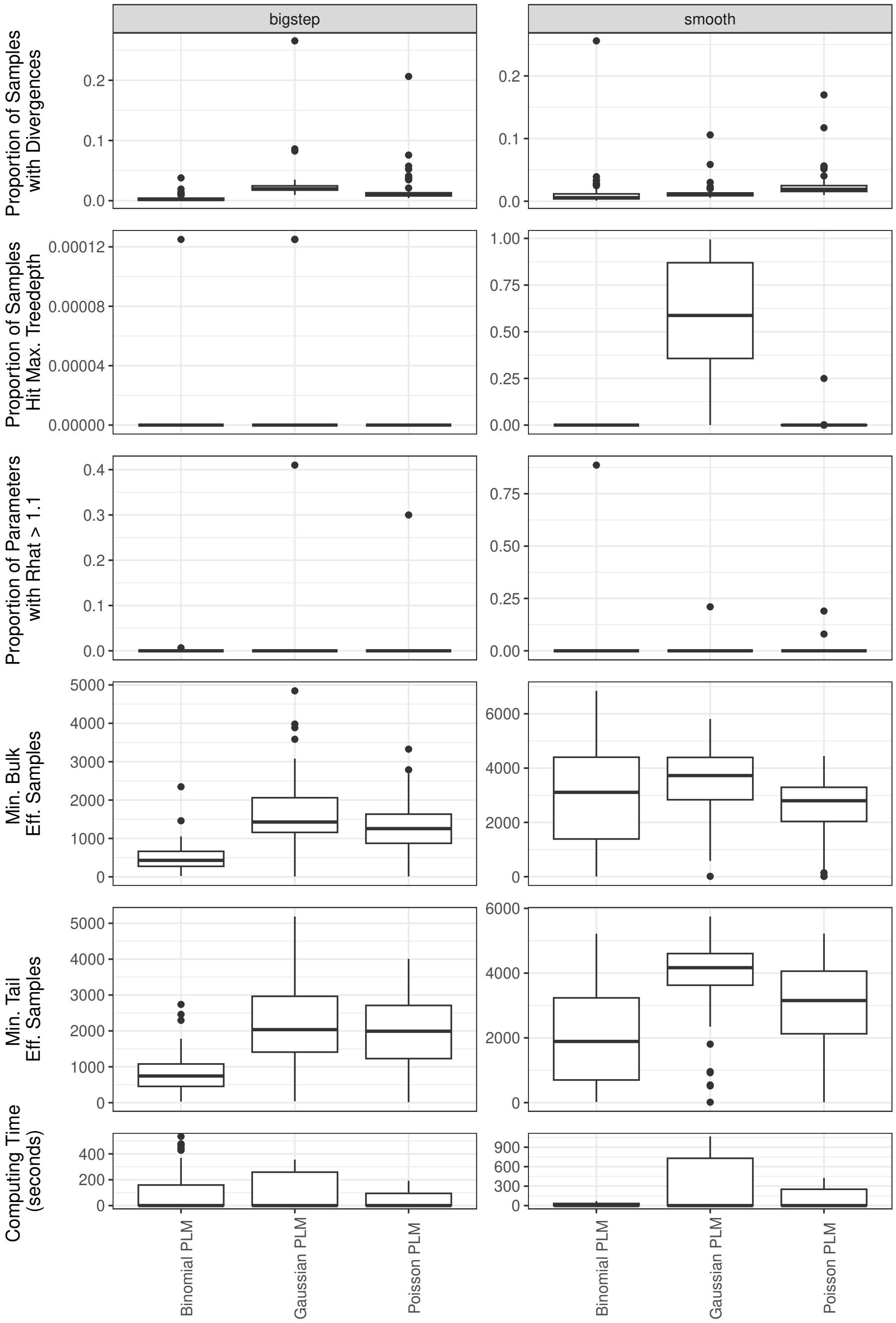

Figure S21: *Computational performance of a horseshoe process regression (HPR) partial linear model, based on 100 replicates in three data-generating scenarios, for continuous, binary, and count outcomes. Smaller is better for all metrics except Min. Bulk. Eff. Samples and Min. Tail. Eff. Samples (the minimum effective sample size in the bulk and tails of the posterior, respectively). Each column is for one data-generating scenario.*

Computational results for the basic HPR simulations are given in Figure S19. Results for the data interpolation simulations are given in Figure S20. Results for the partial linear model simulations are given in Figure S21. Almost all of the models fitted in the simulation studies featured at least some HMC divergences. In most cases less than 5% of samples ended in a divergence. Max treedepth warnings occurred rarely. $\hat{R}$ diagnostics and effective sample sizes generally seemed adequate. Although slow compared to non-Bayesian methods, computation time was generally quite reasonable, with most models finishing in less than 5 minutes.

7 Effects of Hyperparameters and Sample Size

We also explored the role of sample size and prior specification in model estimation. We focused these sensitivity analyses on the bigstep scenario described above, because it is HPR's recommended setting. In addition to the sample size of $n = 100$ that we used above, we also considered $n = 30$ and $n = 500$. We considered several different settings for the hyperparameters of the model:

- The prior mean on the y-intercept α: We recommend setting this hyperparameter to be the sample mean of the data (using appropriate transformations for binary and count outcomes). In the sensitivity analyses below, we compare this approach to 1) setting the prior mean to be the true value of α (alpha_mean = 0) or 2) setting the prior mean to be much larger (alpha_mean = 10).
- The prior standard deviation on the y-intercept α: We recommend setting this hyperparameter to be the sample standard deviation of the data (using appropriate transformations for binary and count outcomes). In the sensitivity analyses below, we compare this approach to 1) setting the prior standard deviation to be too small (alpha_sd = 0.05) or 2) too large (alpha_sd = 50).

- The prior scale c for the global shrinkage parameter τ: We recommend using a value of $c = 0.01$ for this hyperparameter, although in some cases different values may be more suitable. We compare this approach to 1) $c = 1$ and $c = 0.0001$.
- The prior scale s for the measurement error σ, in the case of continuous outcomes: We recommend setting s to be 10 times the sample standard deviation of the data (we used $s = 5$ in these simulations). We compare this approach to 1) setting s to be too small $s = 0.05$ or 2) setting it to be the true value $s = 0.5$.

At each sample size, we generate 100 datasets from the bigstep scenario described in the main manuscript Section 4.1 and in the Supporting Information Section 1. We then run an HPR using all of the recommended hyperparameter settings except the one we are examining (e.g. we would change the value of c but leave the priors on α, σ according to our recommendations). Performance on the metrics of mean absolute difference, credible interval coverage, and credible interval width were compared across hyperparameter settings. We also considered the difference in point estimates and credible interval width between the recommended and alternative settings on each sample dataset.

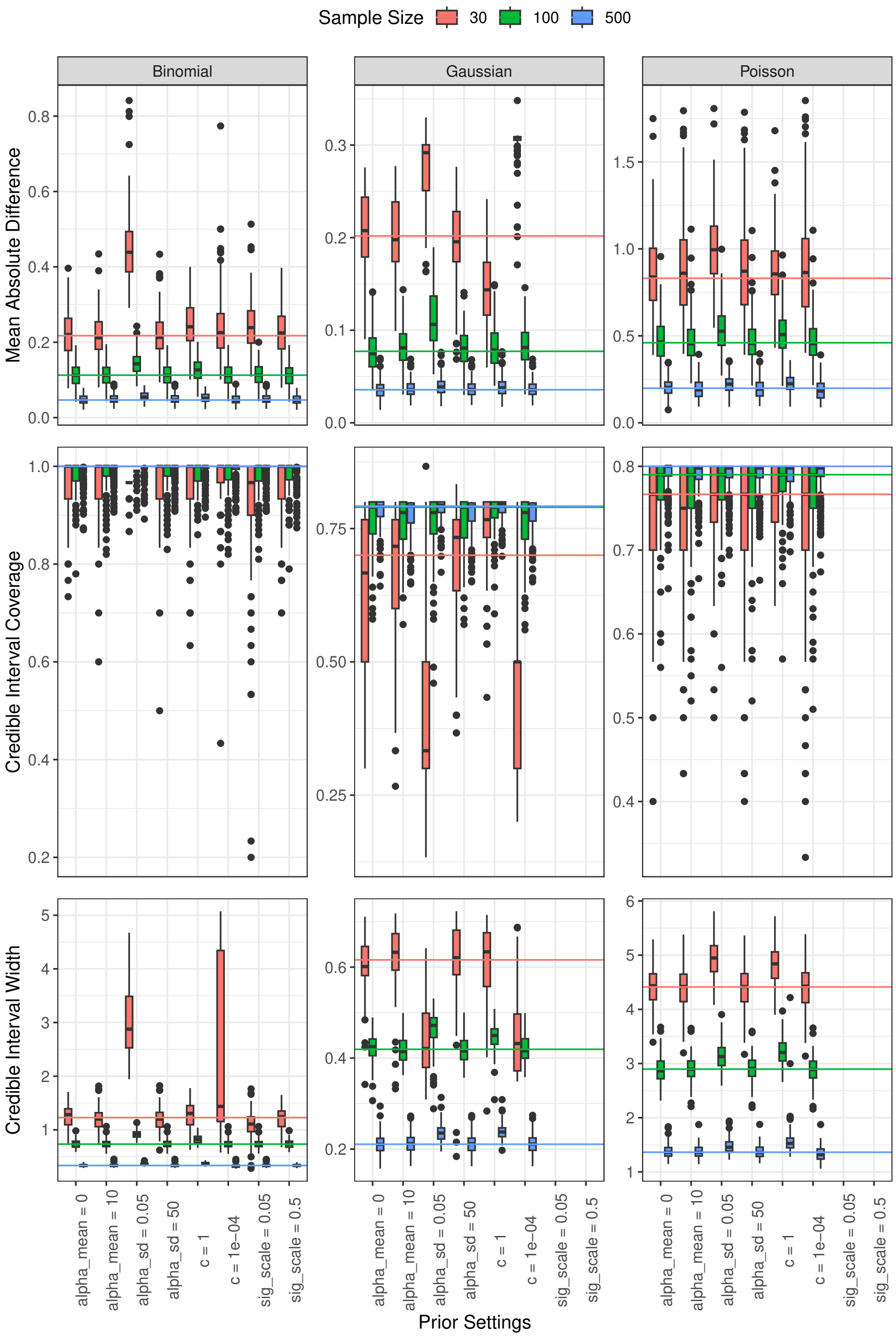

Figure S22: *Sensitivity analyses for the role of hyperparameters and sample size in horseshoe process regression (HPR), based on 100 replicates of the bigstep data generating scenario at three sample sizes ($n = 30, n = 100, n = 500$). The top row gives performance for mean absolute difference (smaller is better); the second row gives performance for credible interval coverage (0.95 is nominal); the third row gives performance for credible/confidence interval width. Each column is for one type of outcome (binary, continuous, and count). Median performance under our recommended hyperparameter settings is given as a horizontal line, with color corresponding to sample size.*

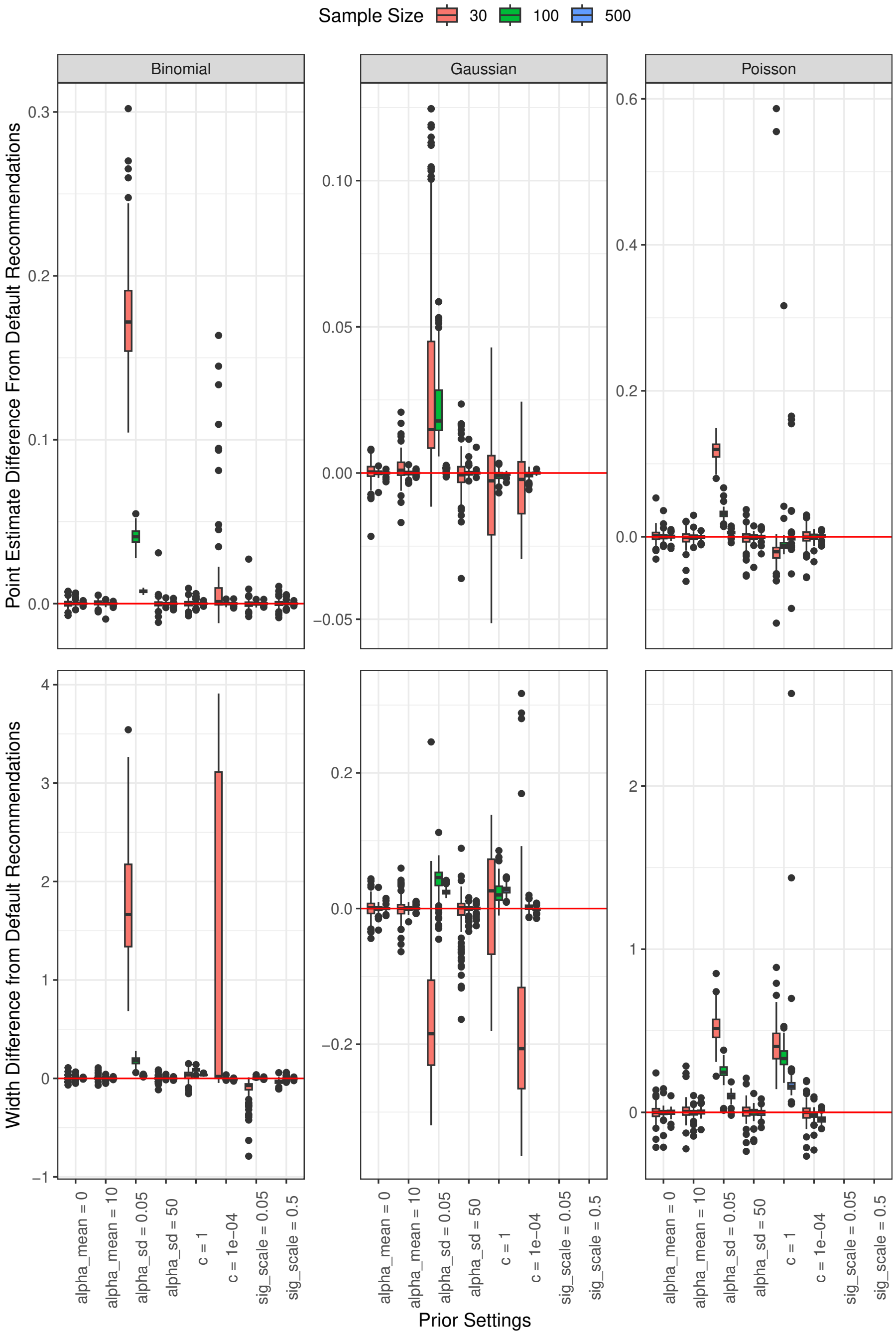

Figure S23: *Sensitivity analyses for the role of hyperparameters and sample size in horseshoe process regression (HPR), based on 100 replicates of the bigstep data generating scenario at three sample sizes ($n = 30, n = 100, n = 500$). The top row gives the difference in point estimates between each alternative hyperparameter choice and our default recommendations, aggregated across timepoints (a difference of 0 is ideal). The second row gives the difference in credible interval width between each alternative hyperparameter choice and our default recommendations, aggregated across timepoints (a difference of 0 is ideal).*

Results are shown in Figures S22 and S23. Performance was generally stable across hyperparameter values, although at smaller sample sizes ($n = 30$), findings were more affected by hyperparameter choices. Poor choices for the prior variance on α–particularly setting it too small–negatively affected model fit. The choice of c also affected findings at small sample sizes, particularly for binary outcomes. Model estimation improved with larger sample sizes, although estimation was still adequate at the $n = 30$ sample size.
